# Structural Basis of Ligand Selectivity by a Bacterial Adhesin Lectin Involved in Multispecies Biofilm Formation

**DOI:** 10.1128/mBio.00130-21

**Published:** 2021-04-06

**Authors:** Shuaiqi Guo, Tyler D. R. Vance, Hossein Zahiri, Robert Eves, Corey Stevens, Jan-Hendrik Hehemann, Silvia Vidal-Melgosa, Peter L. Davies

**Affiliations:** a Department of Biomedical and Molecular Sciences, Queen’s University, Kingston, Ontario, Canada; b Max Planck Institute for Marine Microbiology, Bremen, Germany; University of Washington

**Keywords:** repeats-in-toxin adhesins, bacterial colonization and infection, lectin-carbohydrate interactions, multispecies biofilms, structural biology

## Abstract

Carbohydrate recognition by lectins governs critical host-microbe interactions. *Mp*PA14 (*Marinomonas primoryensis* PA14 domain) lectin is a domain of a 1.5-MDa adhesin responsible for a symbiotic bacterium-diatom interaction in Antarctica. Here, we show that *Mp*PA14 binds various monosaccharides, with l-fucose and *N*-acetylglucosamine being the strongest ligands (dissociation constant [*K_d_*], ∼150 μM). High-resolution structures of *Mp*PA14 with 15 different sugars bound elucidated the molecular basis for the lectin’s apparent binding promiscuity but underlying selectivity. *Mp*PA14 mediates strong Ca^2+^-dependent interactions with the 3,4-diols of l-fucopyranose and glucopyranoses, and it binds other sugars via their specific minor isomers. Thus, *Mp*PA14 only binds polysaccharides like branched glucans and fucoidans with these free end groups. Consistent with our findings, adhesion of *Mp*PA14 to diatom cells was selectively blocked by l-fucose, but not by *N*-acetyl galactosamine. The *Mp*PA14 lectin homolog present in a Vibrio cholerae adhesin was produced and was shown to have the same sugar binding preferences as *Mp*PA14. The pathogen’s lectin was unable to effectively bind the diatom in the presence of fucose, thus demonstrating the antiadhesion strategy of blocking infection via ligand-based antagonists.

## Abstract (precis)

Bacterial adhesins are key virulence factors that are essential for the pathogen-host interaction and biofilm formation that cause most infections. Many of the adhesin-driven cell-cell interactions are mediated by lectins.

## INTRODUCTION

Carbohydrate-based polymers (glycans) are abundant on the exterior of cells (1, 2). The recognition of glycans by carbohydrate-binding proteins, or lectins, underlies many essential biological events such as fertilization, immunological responses, cell-to-cell communications, and host-pathogen interactions (3–9). Some lectins are components of larger adhesion proteins (adhesins), which are key virulence factors that mediate the attachment of bacteria to host cells and other surfaces (6, 10–12). Adhesins further mediate the colonization of bacteria in their favorable niches by helping develop biofilms, which cause over 80% of chronic infections in humans. Once formed, biofilms are resistant to various bactericidal treatments, including the use of antibiotics. With a current shortage of methods to treat biofilm-related diseases and the emerging prevalence of antibiotic-resistant bacteria, there is an urgent need to develop new strategies to treat bacterial infections. Since adhesins play a critical role in the initial stages of biofilm formation, the development of adhesin lectin antagonists holds great promise for treating various infections by blocking bacterial adhesion to human cells. To date, this antiadhesion strategy has led to the development of some promising treatments against diseases. For example, binding of uropathogenic Escherichia coli (UPEC) to mannose-containing glycoproteins of human uroepithelium via the adhesin lectin FimH is an enabling step toward most urinary tract infections (13, 14). Mannoside-based FimH antagonists developed through structure-guided design can effectively block UPEC from binding to the human uroepithelium (15, 16). These compounds have demonstrated fast-acting efficacy against chronic urinary tract infections and can prevent the disease when used as prophylactics (17–19). Despite these successes, widespread application of this antiadhesion approach to treat other bacterial infections is hampered by a lack of knowledge at the molecular level of ligand recognition by other adhesin lectin modules.

Marinomonas primoryensis ice-binding protein (*Mp*IBP) is a large (∼1.5 MDa) repeats-in-toxin (RTX) adhesin found on the surface of its Antarctic Gram-negative bacterium (11, 20–22). While the N terminus of *Mp*IBP anchors the giant protein to the bacterial outer membrane, the ligand-binding modules near the C terminus bind the bacterium to both ice and photosynthetic diatoms to form symbiotic biofilms on the underside of sea ice. *Mp*IBP was initially extracted from the cell lysate of its native bacterium by an ice-affinity purification step (20). Intriguingly, the protein failed to elute from a Superdex S200 size exclusion column intended for further purification. This suggested that the adhesin interacts with the Superdex matrix, which is based on porous agarose particles covalently linked to dextran, a complex branched polymer of α-d-1,6-glucose. Bioinformatic analyses indicated the presence of an ∼20-kDa domain near the C terminus of *Mp*IBP that is a member of the PA14 family, which is a carbohydrate-binding lectin module widely distributed across several kingdoms of life (23, 24). PA14 homologues are found in human proteins like fibrocystin (25) and in fungal and bacterial proteins such as β-glucosidases (26, 27), proteases (23), and adhesins (10, 21, 28, 29). PA14 domains share a β-sandwich fold and the presence of two consecutive aspartate residues in a *cis* peptide linkage (D*cis*D). The D*cis*D motif coordinates a Ca^2+^ ion that is directly involved in binding polar vicinal hydroxyl groups of various carbohydrates (10, 12, 29). Despite these conserved features in their ligand-binding sites, PA14 lectins in microbial adhesins have a broad specificity profile for a range of carbohydrates. In this regard, how PA14 lectins of bacterial adhesins recognize their ligands remains unclear. Yet, this highly conserved module is widespread in adhesins of many different bacteria, including those of human pathogens. These important attributes justify the pursuit of structural studies to elucidate the molecular basis of ligand recognition by *Mp*PA14 (*Marinomonas primoryensis* PA14 domain), which may set the stage for the subsequent development of antagonists to block harmful adhesion.

In this report, we use various types of binding assays and glycan microarrays to show that *Mp*PA14 is a lectin with an unusual binding promiscuity to monosaccharides but is specific in binding certain polysaccharides. X-ray crystal structures of *Mp*PA14 in complex with 15 different simple sugars at atomic resolution reveal the molecular basis for the uncommon ligand selectivity by the lectin. We further show that the adhesion of *Mp*PA14 to its host diatom cells can be fully abolished by a micromolar concentration of l-fucose. Since bioinformatic analyses indicate that lectins highly similar to *Mp*PA14 are present in many bacterial adhesins, including those from human pathogens such as Vibrio cholerae and Vibrio vulnificus (12), there is an opportunity to use a structure-based approach to devise high-affinity lectin antagonists to block harmful biofilm formation or to develop molecular probes to detect these microbes.

## RESULTS AND DISCUSSION

### *Mp*PA14 interacts strongly with fucose and *N*-acetylglucosamine.

**FIG 1:**
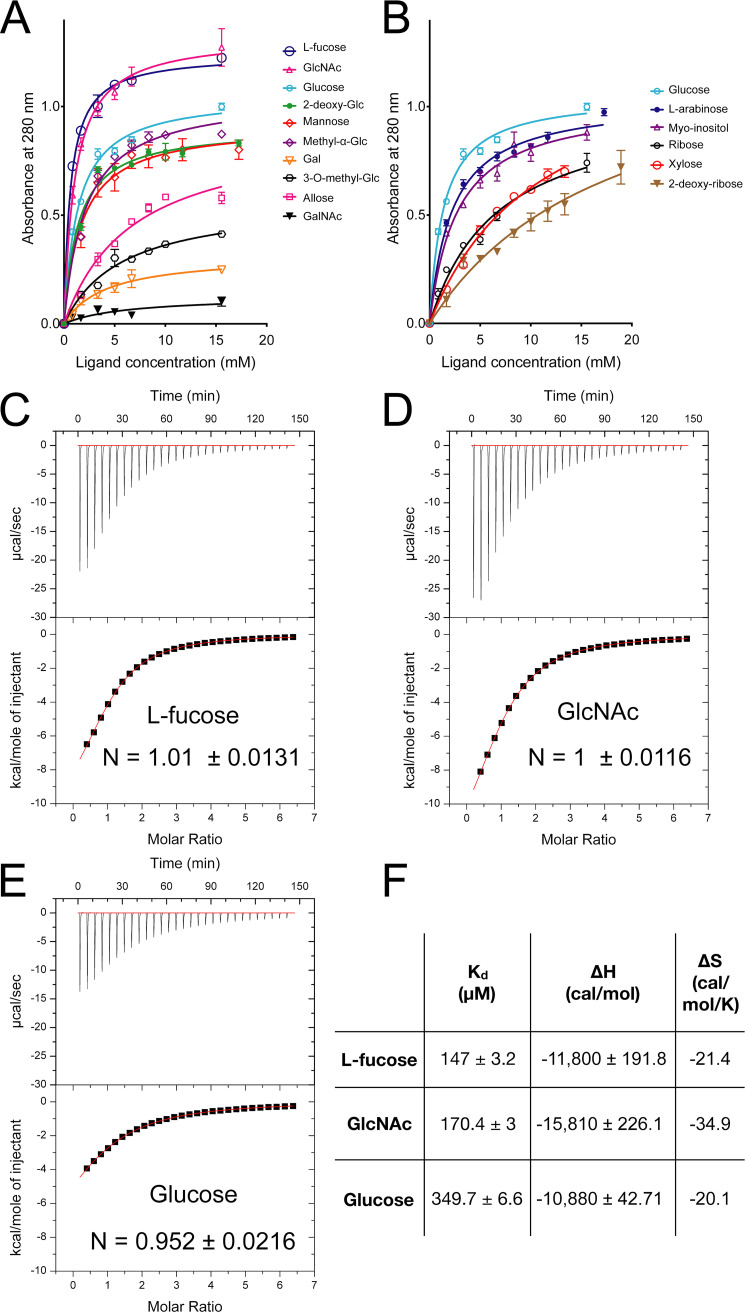
Binding assays to determine relative affinity of *Mp*PA14 for simple carbohydrates. (A) Dextran-based competition binding assay to determine relative affinity of *Mp*PA14 for various hexoses and their derivatives. Each experiment was done in triplicate. (B) Dextran-based competition binding assay to determine relative affinity of *Mp*PA14 for selected pentoses and myo-inositol. Isothermal titration calorimetry of *Mp*PA14 binding to fucose (C), GlcNAc (D), and glucose (E), respectively. The binding stoichiometry (N value) is indicated on each graph. (F) Table of the calculated thermodynamic parameters, including the dissociation constant (*K_d_*), enthalpy (Δ*H*), and entropy (Δ*S*), for each of the three sugars.

To gain insight into the binding specificity of *Mp*PA14, we investigated the relative affinity of various monosaccharides for the lectin by a comparative competition assay (12). *Mp*PA14 bound to Superdex resin was competitively released into solution by the progressive addition of free sugars. The released protein concentrations measured by absorbance at 280 nm were plotted as a function of free sugar concentration to produce semiquantitative binding curves (Fig. 1A and B). The apparent dissociation constant (*K_d_*app) calculated from these binding curves was used as a relative measure of affinity for each assayed sugar (Table 1). *Mp*PA14 lectin bound l-fucose most strongly, with a *K_d_*app of 0.65 mM, followed by *N*-acetylglucosamine (GlcNAc) (*K_d_*app = 1.07 mM; Fig. 1A). Glucose and 2-deoxy-glucose bound the lectin with similar affinity, giving K_d_app values of 1.36 and 1.37 mM, respectively. d-Mannose and methyl-α-d-glucose bound *Mp*PA14 with slightly weaker affinity (*K_d_*app = 1.7 mM and 2.1 mM, respectively), and the binding of d-allose, 3-*O*-methyl-d-glucose and d-galactose was significantly diminished to *K_d_*app values between 4.1 and 6.8 mM. There was no measurable interaction between *Mp*PA14 and *N*-acetyl-galactosamine (GalNAc). d-Ribose bound dextran more strongly than its derivative 2-deoxy-d-ribose (*K_d_*app of 6.8 mM as opposed to 18 mM; Fig. 1B and Table 1). l-Arabinose exhibited higher affinity than the other pentoses for *Mp*PA14, with a *K_d_*app of 2.2 mM.

**TABLE 1:** Apparent dissociation constants for the binding of different sugars

Sugar	*K_d_*app (mM)
l-Fucose	0.65
*N*-acetyl-glucosamine	1.07
Glucose	1.4
2-Deoxy-glucose	1.4
Mannose	1.7
Methyl-α-glucose	2.1
l-Arabinose	2.2
Inositol	2.7
Galactose	4.1
3-*O*-methyl-glucose	5.7
Allose	6.8
Ribose	6.8
Xylose	11
2-Deoxy-ribose	18
*N*-acetyl-galactosamine	–^*a*^

a No detectable binding was observed for *N*-acetyl-galactosamine.

To further assess the binding thermodynamic parameters of *Mp*PA14 ligands, we performed isothermal titration calorimetry (ITC) with l-fucose, *N*-acetylglucosamine (GlcNAc) and glucose (Fig. 1C to E). The ITC measurements produced rectangular hyperbolic curves for all three simple saccharides, and fitted well to a one-binding-site model with calculated binding stoichiometry (N) values being close to 1 (12, 21). ITC ranked the affinity of these three ligands in the same order as that shown by the competition binding assay, with l-fucose as the strongest ligand, followed by GlcNAc and then glucose. The dissociation constant (*K_d_*) values calculated from the ITC measurements were significantly lower than the *K_d_*app values obtained from the competition assay (Fig. 1F and Table 1). However, this is to be expected, because the dextran beads used in the competition assay have multiple binding sites nearby that attract the lectin, whereas the calorimetry was done with free sugars in solution. With *K_d_* values of 147 μM and 170 μM for l-fucose and GlcNAc, these two ligands had a greater than 2-fold higher affinity for *Mp*PA14 than did glucose (*K_d_* = 350 μM). In general, the affinity of lectins for monosaccharides (*K_d_* values) lies within the high micromolar to millimolar range (8, 30, 31). Thus, our results showed the *Mp*PA14 domain from its bacterial adhesin had relatively high affinity for the three strongest ligands in comparison to other lectins. Furthermore, negative enthalpy (Δ*H*) and entropy (Δ*S*) contributions were calculated for all three carbohydrates when they bound to *Mp*PA14, which indicated the binding was driven primarily by polar interaction such as the formation of hydrogen and ionic bonds rather than by hydrophobic interactions. This was consistent with the observation that the Ca^2+^-dependent ligand-binding site of *Mp*PA14 consisted of mainly polar and charged amino acids without any residues with large hydrophobic side chains. We therefore acquired detailed structural information to study *Mp*PA*14* ligand recognition.

### Structural basis of *Mp*PA14 selectivity for glucopyranoses.

To examine the molecular basis of carbohydrate recognition by *Mp*PA14, we determined the X-ray crystal structures of the lectin in complex with 14 new saccharides to a resolution of 1 to 1.3 Å (see Table S1 to S3 in the supplemental material). The lectin fold is a β-sandwich domain that binds four to seven Ca^2+^ ions (Ca1 to Ca7) on its surface (21) (see Fig. S1A in the supplemental material. Ca1 is coordinated by the D*cis*D motif (Asp110 and Asp111) on the periphery of the protein, which is directly involved in binding carbohydrate with help from amino acid residues in loops 9 and 11 (L9 and L11; Fig. S1B). While Ca2 to Ca4 likely have a role in lectin folding (12, 21) (Fig. S1C to E), Ca5-7 have few ligands from the lectin and are probably an artifact from the crystallization condition that contained over 100 mM CaCl_2_. The highly electronegative surface of *Mp*PA14 is consistent with its capacity to bind a high number of Ca^2+^ ions (Fig. S1F). There were no substantial conformational changes to the overall lectin fold when it was complexed with different sugars (root mean square deviation [RMSD] < 0.1 Å).

10.1128/mBio.00130-21.1 FIG S1 Structural features of *Mp*PA14. (A) Overview of the *Mp*PA14 structure in complex with glucose. (Right) Interface from a view that is rotated approximately 180° around a vertical axis from the left. Carbon atoms are colored grey, oxygen atoms are red, and nitrogen atoms are blue. The glucose-coordinating Ca1 is shown as a dark blue sphere, while the other Ca^2+^ ions are shown as green spheres. (B) Zoomed-in view of the ligand-binding site of *Mp*PA14. The D*cis*D motif is indicated by an arrow. (C to E) Ca^2+^ coordination sites for Ca2 to Ca4. (F) Surface electrostatic potential of *Mp*PA14. Surfaces that are electronegative are colored in red, while those that are neutral are colored white, and the electropositive surfaces are colored blue. (G) Amino acid alignment of PA14 domains from Marinomonas primoryensis (*Mp*PA14), the oil-eating bacterium Marinobacter hydrocarbonoclasticus (*Mh*PA14), and the cholera-causing human pathogen Vibrio cholerae (*Vc*PA14). Amino acid residue number and secondary structure components for *Mp*PA14 are indicated. Conserved residues that constitute the carbohydrate-binding site are highlighted in magenta. Red arrowhead points to the D*cis*D motif. Loops (L9 and L11) involved in carbohydrate binding are indicated in red. Download FIG S1, PDF file, 0.3 MB. Copyright © 2021 Guo et al. 2021 Guo et al. https://creativecommons.org/licenses/by/4.0/ This content is distributed under the terms of the Creative Commons Attribution 4.0 International license.

10.1128/mBio.00130-21.7 TABLE S1 X-ray crystallographic statistics for *Mp*PA14 in complex with l-fucose, mannose, α-methyl-glucose, inositol, GlcNAc, allose, and 3-*O*-methyl glucose. Download Table S1, DOCX file, 0.02 MB. Copyright © 2021 Guo et al. 2021 Guo et al. https://creativecommons.org/licenses/by/4.0/ This content is distributed under the terms of the Creative Commons Attribution 4.0 International license.

**FIG 2:**
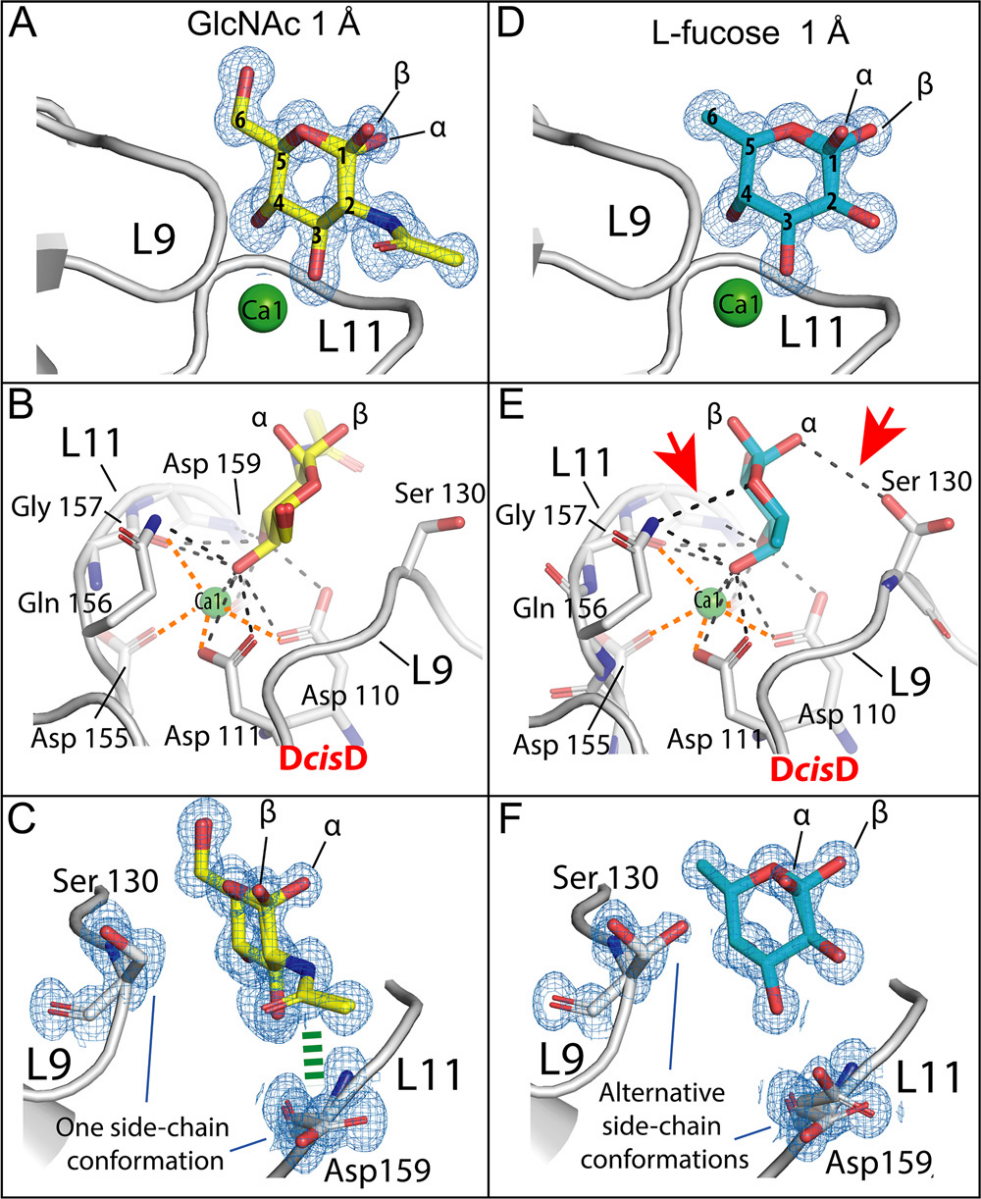
Ligand-binding site of *Mp*PA14 in complex with GlcNAc and l-fucose. Structural features of the *Mp*PA14 ligand-binding sites with GlcNAc (A) and l-fucose (D). Detailed carbohydrate-protein interactions between *Mp*PA14 and GlcNAc (B), and l-fucose (E), respectively. Ionic bonds (∼2.5 Å) between Ca1 and *Mp*PA14 are indicated by thicker orange dashed lines, while polar interactions between the lectin and carbohydrates are indicated with black dashed lines (shorter than 3.5 Å). Red arrowheads in panel E indicate the additional polar interactions between l-fucose and *Mp*PA14 compared to those in panel B. (C, F) Angled view comparing the conformations of the side chains of L9-Ser130, and L11-Asp159 in the ligand-binding sites of *Mp*PA14-GlcNAc (C) and *Mp*PA14-fucose (F) complexes. Carbon atoms of GlcNAc are colored yellow, while those of l-fucose and *Mp*PA14 are colored cyan and light gray, respectively. The interaction between the acetyl group of GlcNAc and side chain of Asp159 is indicated by a green dashed line (∼4 Å). Oxygens are colored red, nitrogens are colored blue, and Ca^2+^ ions are colored green. (A, C, D, and F) The 2 *F*_o_ − *F*_c_ maps shown for sugars and ligand-binding site residues are shown as blue meshes (contoured at σ = 1).

As suggested by ITC, sugar recognition by *Mp*PA14 is primarily driven by polar interactions in a Ca^2+^-dependent manner. Glucose, GlcNAc, and other glucopyranose-containing carbohydrates, including 2-deoxy-glucose, methyl-α-glucose, and two disaccharides (sucrose and trehalose), all bound to the *Mp*-PA14-Ca1 via their *trans* vicinal 3,4-diols (ionic bond length, 2.5 Å) in gauche configuration with a dihedral angle of ∼60° (Fig. 2A; see also Fig. S2 and Fig. S3A and B in the supplemental material). This interaction is further enhanced with the diol being coordinated by the side chain carboxyl and hydroxyl oxygens of the D*cis*D motif, and main-chain and side chain protein ligands from L11 (Gln156, Gly157, and Asp159; Fig. 2B). The acetyl group on the C-2 position of GlcNAc interacts with the side chain atoms of Asp159 on L11 (Fig. 2C), holding the aspartate side chain down in one stable conformation. This additional interaction likely accounts for the higher affinity of GlcNAc to the lectin than that of the other glucopyranoses.

10.1128/mBio.00130-21.2 FIG S2 Structures of *Mp*PA14 in complex with glucopyranose-containing sugars. The color scheme and labeling are the same as those in Fig. 2. Download FIG S2, PDF file, 0.2 MB. Copyright © 2021 Guo et al. 2021 Guo et al. https://creativecommons.org/licenses/by/4.0/ This content is distributed under the terms of the Creative Commons Attribution 4.0 International license.

**FIG 3:**
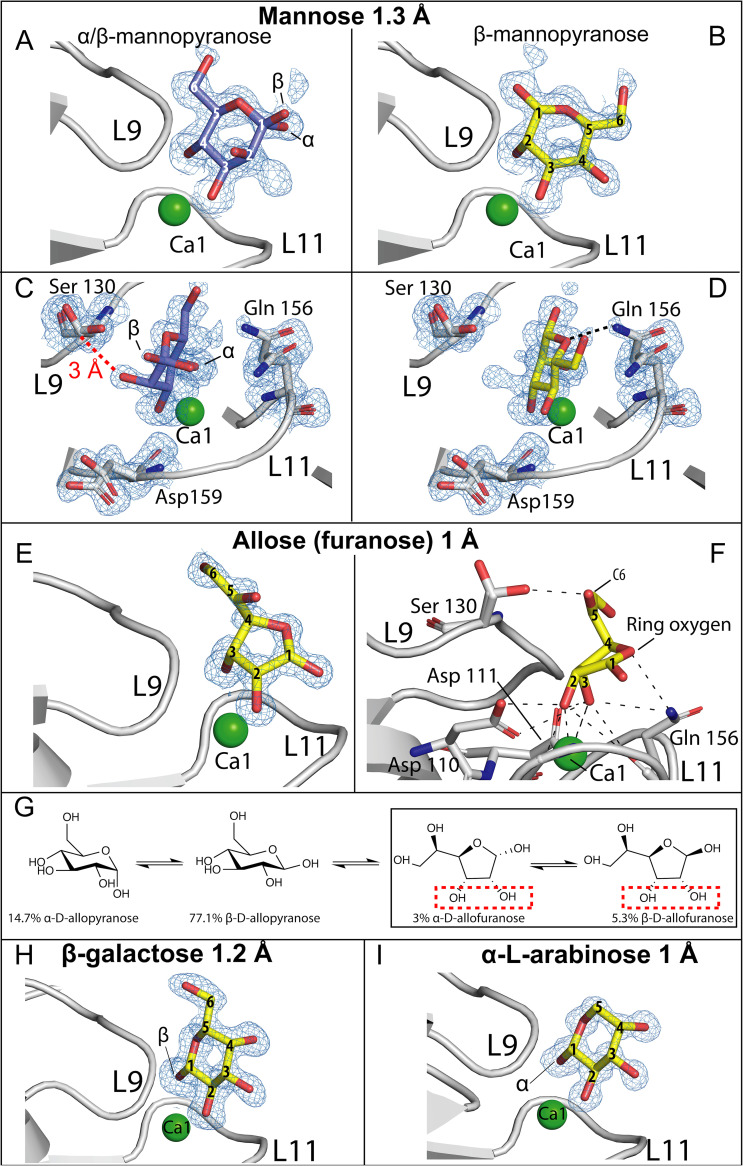
Ligand-binding site of *Mp*PA14 in complex with mannose, allose, galactose, and l-arabinose. Side (A) and top views (C) of α- and β-mannopyranose rings anchored to *Mp*PA14 via their 3,4-diols. Electron density for only β-mannopyranose was seen for the binding mode via the 2,3-diol. (B) Side view; (D) top-down view. The distance between the β-carbon of serine on L9 and C-2 hydroxyl oxygen of α/β-mannopyranose is indicated by a red dashed line. (E) Side-view of β-allofuranose in the *Mp*PA14 ligand-binding site. (F) Detailed polar interactions (black dashed lines) between β-allofuranose and *Mp*PA14. Amino acid residues involved in allose-*Mp*PA14 interaction are labeled. The color scheme is the same as that in Fig. 3. (G) Equilibria of allose anomers in aqueous solution at 30°C. The diol of allofuranose responsible for binding *Mp*PA14 is indicated by a red dashed box. Side-view of β-galactose (H) and α-l-arabinose (I) in the *Mp*PA14 ligand-binding site.

10.1128/mBio.00130-21.3 FIG S3 Structures of strongly binding (A), moderately binding (B), and weakly binding (C) sugars of *Mp*PA14. The specific isomer shown for each monosaccharide was revealed by X-ray crystallography as described in Fig. 2 and 3 and Fig. S2 and S5. Double arrowheads point to the diol groups used by the sugar for interaction with Ca1 in the ligand-binding site of *Mp*PA14. Alternative binding configurations are indicated with double arrowheads in blue or green instead of red. Download FIG S3, PDF file, 0.1 MB. Copyright © 2021 Guo et al. 2021 Guo et al. https://creativecommons.org/licenses/by/4.0/ This content is distributed under the terms of the Creative Commons Attribution 4.0 International license.

Structural data shown here for *Mp*PA14 contrast with the previously reported structure of *Mh*PA14 in complex with glucose (12). *Mh*PA14 is an *Mp*PA14 homolog from an RTX adhesin of the oil-degrading bacterium Marinobacter hydrocarbonoclasticus. With a similar ligand-binding site to that of *Mp*PA14 (12), *Mh*PA14 also had a strong preference for binding l-fucose and glucopyranoses over other monosaccharides. However, X-ray crystallography showed *Mh*PA14 complexing glucopyranose via its 1,2-diol. Given that the C-2 position of GlcNAc lacks the hydroxyl group required for interacting with *Mp*PA14 via the 1,2-diol, the binding mode shown by the *Mh*PA14-glucose complex could not explain the lectin’s high affinity for this acetylated sugar. Close inspection of the *Mh*PA14-glucose complex structure revealed that the monosaccharide in the carbohydrate-binding site is in direct contact with a neighboring symmetry-related molecule (see Fig. S4A and B in the supplemental material), indicating the tight packing of the unit cell caused the sugar to bind in a less favorable configuration. The observed crystal-packing artifacts explained why cocrystallization of *Mh*PA14 with various other sugars, and direct soaking experiments with the apo-*Mh*PA14 crystal, were futile. In contrast, symmetry-related molecules in the unit cells of *Mp*PA14 are far apart from the ligand-binding site and thus do not impact the binding of monosaccharides to *Mp*PA14 (Fig. S4C and D).

10.1128/mBio.00130-21.4 FIG S4 Crystal packing of *Mh*PA14 and *Mp*PA14. (A) Crystal unit cell of *Mh*PA14 in complex with glucose. The box with black dashed lines indicates the interface between one molecule of *Mh*PA14-glucose (grey) and its neighboring symmetry-related molecules (magenta) in the crystal unit cell. (B) The glucose molecule bound by *Mh*PA14 is directly involved in crystal contact with a symmetry-related molecule. Lengths for the protein-sugar interaction bonds (black dashed lines) are indicated. (C) Crystal unit cell of *Mp*PA14 in complex with GlcNAc. The box with black dashed lines indicates the interface between one molecule of *Mp*PA14-GlcNAc (grey) and its neighboring symmetry-related molecules (magenta) in the crystal unit cell. (D) The GlcNAc bound by *Mp*PA14 does not interact with a symmetry-related molecule in the crystal unit cell. The distances between the GlcNAc and the nearest atoms from a neighboring symmetry-related molecule are indicated by green dashed lines. Download FIG S4, PDF file, 0.3 MB. Copyright © 2021 Guo et al. 2021 Guo et al. https://creativecommons.org/licenses/by/4.0/ This content is distributed under the terms of the Creative Commons Attribution 4.0 International license.

### Why l-fucose is a better ligand than glucopyranoses.

Based on results from docking experiments, it was proposed that l-fucose binds *Mh*PA14 via its 2,3-diol (12). However, the well-resolved 1-Å electron density map in this study unambiguously showed that fucose bound *Mp*PA14 with its *cis* 3,4-diol in the gauche conformation with a dihedral angle of 47° (Fig. 2D). In contrast to the *Mp*PA14 hexose ligands, which are all in the d-configuration, fucose is in the l-configuration, with hydroxyl groups of the fucopyranose ring pointed in opposite directions. This helps the endocyclic oxygen atom of fucose point toward L11 and hydrogen bond with the sidechain of Gln156 (Fig. 2E). Additionally, the hydroxyl on the α-anomeric carbon may hydrogen bond with the sidechain of Ser130 on L9, clamping the pyranose ring tightly into the binding site (Fig. 2E and F). The l-fucose–*Mp*PA14 interaction is distinct from that shown by the bacterial C-type lectin, LecB, from Pseudomonas aeruginosa, which uses two side-by-side Ca^2+^ ions to directly coordinate the 2,3,4-triol of l-fucose (32, 33). The additional ionic interaction between LecB and l-fucose can explain its enhanced affinity (*K_d_* = 58 μM) for the sugar compared to that of *Mp*PA14 (*K_d_* = 147 μM).

### Promiscuity of *Mp*PA14 in monosaccharide recognition.

To investigate the molecular basis for the plastic nature of *Mp*PA14 in binding various monosaccharides, we investigated *Mp*PA14 structures in complex with various glucose epimers and derivatives.

Mannose bound *Mp*PA14 slightly more weakly than glucose (Fig. 1A and Table 1). The electron density map for the mannose-*Mp*PA14 complex indicated that the sugar bound in two distinct conformations (Fig. 3A and B; see also Fig. S3B). Like glucose, d-mannose bound *Mp*PA14 via the 3,4-diol. However, since the mannose C-2 hydroxyl moiety is in an axial position, its oxygen atom may clash with the β-carbon of the Ser130, as they are only 3 Å away from each other (Fig. 3C). In addition, as the C-2 and C-3 hydroxyl groups of mannose are positioned in *cis*, they may form an intramolecular hydrogen bond that further weakens the 3,4-diol from binding Ca1. Alternatively, β-mannopyranose can bind *Mp*PA14 using a second configuration where its 2,3-diol anchors the saccharide ring in an inverted fashion, allowing the ring oxygen to hydrogen bond with the side chain amide group of the Gln156 (Fig. 3D). However, α-mannopyranose failed to fit into the electron density via this binding mode, which indicates that *Mp*PA14 can only recognize the less prevalent β-anomer in the equilibrium via its 2,3-diol (33% β-mannopyranose as opposed to 62% α-mannopyranose at 30°C) (34). This apparent lack of one distinct stable binding configuration explains the relatively inferior affinity of mannose compared to glucose.

With the C-3 hydroxyl in the axial position, allopyranose cannot bind *Mp*PA14 via its *cis* vicinal 3,4-diol, as this would cause the saccharide ring to clash with residues of L11 (e.g., Gln156). Unexpectedly, the binding site of the lectin contained the furanose form of allose, with its 2,3-diol interacting with Ca1 (Fig. 3E and Fig. S3C). The furanose rings lean toward L11, with their endocyclic oxygen interacting with the side chain amide group of Gln156, and the C-5 hydroxyl group extends out to hydrogen bond with the side chain hydroxyl of Ser130 (Fig. 3F). At 30°C, approximately 92% of allose exists as pyranoses in solution (Fig. 3G), yet *Mp*PA14 binding appears to be dependent on the rare presence of the allofuranose, which only makes up ∼8% of allose in solution, explaining the feeble affinity of this sugar for the lectin (Table 1). Similarly, 3-*O*-methyl-glucose cannot bind *Mp*PA14 via the 3,4-diol because of the substituted methyl group on its C-3. This sugar compensates by binding using the 1,2-diol of the β-anomer (Fig. S3C), resulting in its considerably weaker binding to the lectin compared to that of glucopyranoses and mannose.

Galactose has its C-4 hydroxyl in the axial position instead of being equatorial as in glucose. Docking of the galactopyranose ring to *Mp*PA14 via the 3,4-diol is not possible due to steric hinderance against Gln129 and Ser130 on L-9. Instead, galactose can only interact with *Mp*PA14 via the 1,2-diol of its rare β-anomer (Fig. 3H). These limitations explain the relatively weak interaction between galactose and *Mp*PA14 (Table 1). Results from the binding analysis of GalNAc verify this assessment. Having just shown the 3,4-diol of galactopyranose cannot complex *Mp*PA14, binding of GalNAc is completely abolished because its 1,2-diol is unavailable due to the C-2 hydroxyl being substituted with an acetyl group.

As a comparison to *Mp*PA14 and its close homologs, C-type lectins also bind monosaccharide vicinal hydroxyl groups via Ca^2+^. In addition, sugar selectivity by C-type lectins typically involves interactions through amino acid residues with aromatic sidechains. For instance, the C-type lectins that selectively bind galactose require the presence of a tryptophan that aligns with the open face of the pyranose ring akin to a hydrophobic stacking interaction (or histidine that selects for mannose [35, 36]). This interaction with hydrophobic amino acids is common not only in lectins but also in other carbohydrate-binding modules (8) and in carbohydrate-active enzymes (37), where it often imparts stringent selectivity for the sugar type bound. Sometimes multiple aromatic side chains situated along the glycan binding site contribute to the recognition of multiple monomers in a glycan, further increasing selectivity not only for the monomers but also for how they are linked. *Mp*PA14 lacks these hydrophobic residues in its interactions with only the terminal sugars. As a result, the relatively more flexible ligand-binding site of *Mp*PA14 may contribute to its higher degree of plasticity to engage sugar monomers that vary in stereochemistry.

We further analyzed the crystal structures of *Mp*PA14 in complex with three pentoses, l-arabinose, ribose, and 2-deoxy-ribose, as well as inositol, which has an unusual 6-carbon saccharide ring without an endocyclic oxygen (see Fig. S3B and C and Fig. S5 in the supplemental material). These four carbohydrates bound *Mp*PA14 more weakly than glucose and mannose (Table 1). Crystal structures showed that *Mp*PA14 selects the pyranose form of pentoses for binding. As pentoses have a higher percentage of furanose present in the conformational equilibria than do hexoses, selectivity for the more thermodynamically stable pyranoses might contribute to the weaker affinity of pentoses toward the lectin. For instance, despite l-arabinose existing in solution predominantly in the pyranose form at 25°C (57% α-arabinopyranose versus 30.5% β-arabinopyranose) (38), only the α-anomer complexes *Mp*PA14 via its 1,2-diol (Fig. 3I). The same rationale can be used to explain the even weaker affinity of ribose and its derivative 2-deoxy ribose for *Mp*PA14 (se Fig. S5B and C in the supplemental material) (39). In the case of inositol, the composite electron density map indicates it binds *Mp*PA14 in several different conformations (Fig. S5D to F). This promiscuous binding mode is indicative of a lack of one stable binding conformation, which might explain inositol’s moderate affinity for *Mp*PA14.

10.1128/mBio.00130-21.5 FIG S5 Structures of *Mp*PA14 in complex with its moderately and weakly binding ligands. (A) β-3-*O*-Methyl-glucose. (B) Ribose. (C) 2-deoxy-ribose. (D to F) Three binding conformations of Inositol. The color scheme and labeling are the same as those in in Fig. S2. Download FIG S5, PDF file, 0.2 MB. Copyright © 2021 Guo et al. 2021 Guo et al. https://creativecommons.org/licenses/by/4.0/ This content is distributed under the terms of the Creative Commons Attribution 4.0 International license.

In summary, X-ray crystallography has elucidated the molecular basis of *Mp*PA14’s promiscuity in binding a range of monosaccharides. Remarkably, the lectin can discern favorable conformations of these monosaccharides from their nonbinding anomers, even when the latter are much more prevalent in the equilibria (e.g., allofuranose as opposed to allopyranose). Nevertheless, monovalent or simple carbohydrate oligomers are typically not the physiological targets of lectins (30). In the context of *Mp*PA14, the lectin likely binds complex glycans or glycoproteins present on the surfaces of microbes, where the proximity effect of having numerous identical or similar end groups increases the avidity of the lectin interaction. In this way the sugar-binding activity of *Mp*PA14 can help to form biofilms. This prompted us to survey the lectin-sugar interactions from a broad spectrum of complex carbohydrates using glycan microarray technology.

### *Mp*PA14 binds glucopyranose and fucose moieties of complex glycans.

**FIG 4:**
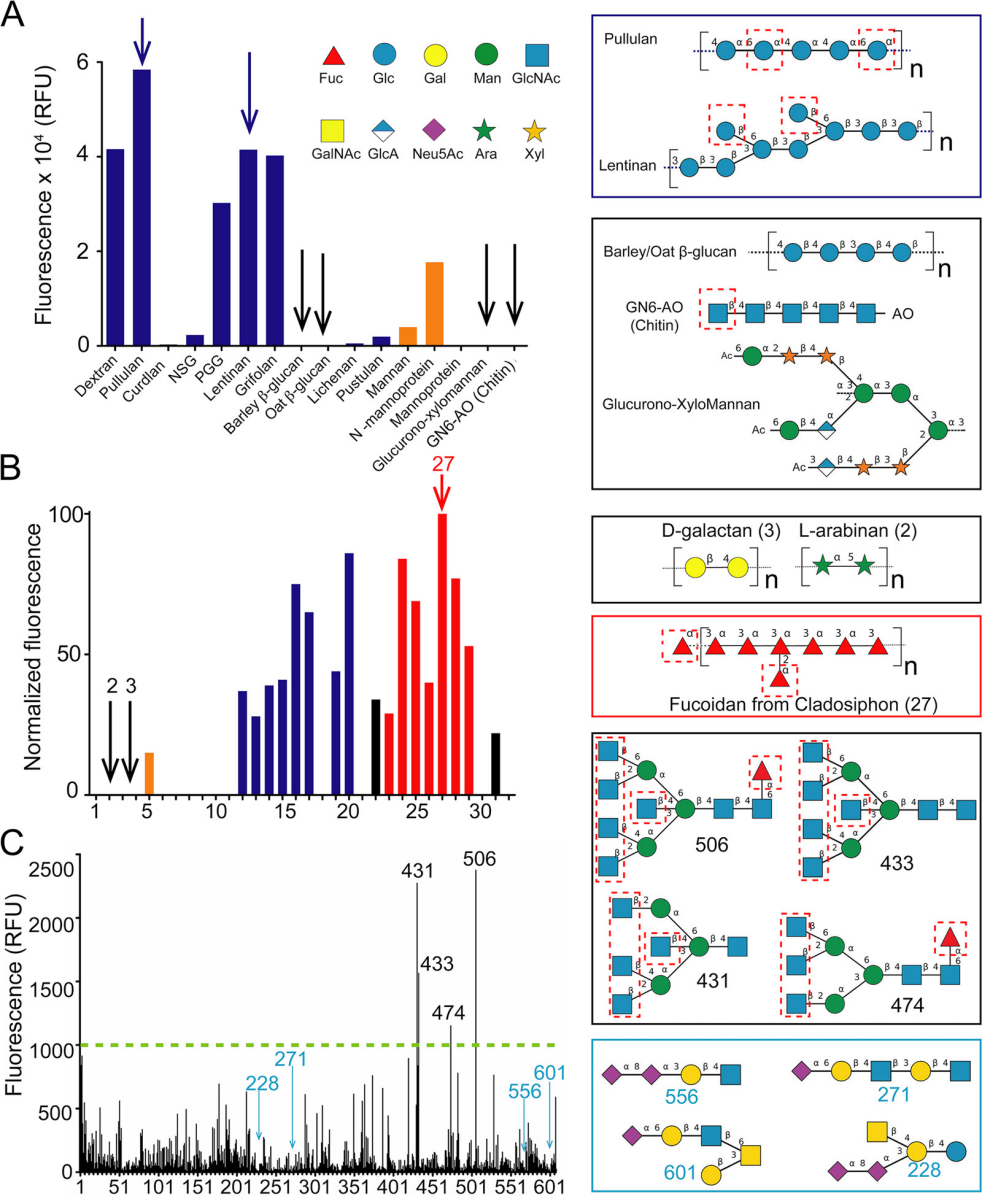
Binding of *Mp*PA14 to sugar oligomers identified by three different glycan microarrays. (A) Microbial glycan array analysis conducted at the Imperial College Glycosciences Laboratory. Data bars showing binding intensity for glucans and mannans are colored blue and orange, respectively. Examples of strong binders are drawn in the blue box (top right, blue arrows in the graph), while those for weak binders are drawn below (black arrows in the graph). The putative sites for *Mp*PA14 binding to the glycans are indicated by dashed red boxes. Glycan nomenclature symbols are included. Abbreviations are as follows: Fuc, l-fucose; Glc, glucose; Gal, galactose; Man, mannose; GlcNAc, *N*-acetylglucosamine; GalNAc, *N*-acetyl-galactosamine; GlcA, glucuronic acid; Neu5Ac, *N*-acetyl-neuraminic acid; Ara, l-arabinose; Xyl, xylose. (B) Microarray data of glycans from marine algae and land plants. Glucans (blue), mannans (orange) fucoidans (red), and other types of glycans (black). Examples of nonbinders (right, black arrows in the graph), while the structure for Cladosiphon fucoidan is shown below (red arrow in the graph). (C) Mammalian glycan array analyzed at the Consortium for Functional Glycomics. Representative structures for the strong *Mp*PA14 binders (black box) and nonbinders (blue box) are drawn on the right.

To dissect *Mp*PA14’s role in the formation of mixed-species biofilms, we probed two different microbial glycan microarrays for lectin-binding partners (40). We first analyzed the binding of GFP-*Mp*PA14 to 16 different polysaccharides consisting primarily of glucans and mannans from bacteria and fungi (Imperial College Glycosciences Laboratory). Four glucans, namely pullulan, lentinan, dextran, and grifolan, bound most avidly to *Mp*PA14 (Fig. 4A). To emphasize the specificity of this interaction, six of the other polysaccharides showed negligible or no detectable binding; these included glucans such as curdlan and those purified from oat and barley, as well as mannoprotein, glucurono-xylomannan, and GN6-AO, which is a hexasaccharide of 1,4-linked GlcNAc (Chitin) with an aminooxy group (AO; see Table S4 in the supplemental material. Consistent with findings from our structural and binding analyses (Fig. 1 and 3), the four strongest binders contain multiple glucopyranoses with unoccupied 3,4-diols either as internal or terminal moieties in their linear backbone (pullulan) as well as in their branches (lentinan, dextran, and grifolan; Fig. 4A). In contrast, weak or negligible binding to *Mp*PA14 was demonstrated for linear glucans formed through 1,3 and 1,4 linkages, such as β-glucans of oat and barley. Each of these linear polysaccharides has only one 3,4-diol set from its terminal glucopyranose accessible for *Mp*PA14 binding, while all potential 3,4-diols in the backbone are involved in the formation of glycosidic bonds (Fig. 4A). Similarly, GN6-AO interacted poorly with *Mp*PA14 because it too has only one free 3,4-diol in the terminal GlcNAc. Furthermore, moderate lectin-glycan interactions were observed for the highly branched *N*-mannoprotein from the fungus Candida albicans (Fig. 4A, orange bar; see also Table S4), while binding to glucurono-xylomannan and mannoprotein from the fungus Aspergillus fumigatus was negligible, as these glycans lack the more favorable structural epitopes of 3,4-diols on either glucopyranose or l-fucose.

10.1128/mBio.00130-21.8 TABLE S2 X-ray crystallographic statistics for *Mp*PA14 in complex with 2-deoxy-glucose, galactose, l-arabinose, ribose, 2-deoxy-ribose, sucrose, and trehalose. Download Table S2, DOCX file, 0.02 MB. Copyright © 2021 Guo et al. 2021 Guo et al. https://creativecommons.org/licenses/by/4.0/ This content is distributed under the terms of the Creative Commons Attribution 4.0 International license.

10.1128/mBio.00130-21.9 TABLE S3 Polysaccharides used in the first glycan array. The first column on the left presents the relative lectin-binding activity detected. Download Table S3, DOCX file, 0.02 MB. Copyright © 2021 Guo et al. 2021 Guo et al. https://creativecommons.org/licenses/by/4.0/ This content is distributed under the terms of the Creative Commons Attribution 4.0 International license.

10.1128/mBio.00130-21.10 TABLE S4 Polysaccharides contained in the second glycan array. The first column on the left presents the lectin-binding activity detected (normalized GFP-*Mp*PA14 signal intensity). Polysaccharide structures are listed in the same order as in the *x* axis from Fig. 4B (order of appearance shown in second column). Download Table S4, DOCX file, 0.02 MB. Copyright © 2021 Guo et al. 2021 Guo et al. https://creativecommons.org/licenses/by/4.0/ This content is distributed under the terms of the Creative Commons Attribution 4.0 International license.

To expand the repertoire of glycans beyond glucans and mannans, we performed a second focused microarray composed of 32 polysaccharides found in microbes such as fungi and bacteria, as well as in macroalgae and plants (Fig. 4B). Similarly to the first microarray, *Mp*PA14 showed significant binding to some glucans that present multiple sets of unoccupied 3,4-diols, which included pustulan, pachyman, and scleroglucan (Fig. 4B, dark blue bars, glycans 20, 16 and 17, respectively; see also Table S4 in the supplemental material). In addition, *Mp*PA14 interacted avidly with fucoidans from macroalgae. The strongest *Mp*PA14-binding fucoidans were to Cladosiphon (41), Sargassum, and Ascophyllum nodosum (glycans 27, 24, and 28, red bars). Since fucoidans are algal polysaccharides primarily consisting of a linear backbone of sulfated α-1,3- or α-1,4-linked l-fucose (42, 43), *Mp*PA14 can interact by binding the terminal moieties on the backbone and branches with unoccupied 3,4-diols. In contrast, *Mp*PA14 did not bind to polysaccharides such as arabinan, galactan, galactomannan, xylan, xyloglucan, and porphyran (glycans 2, 3, 6, 11, 21, and 32, respectively; Fig. 4B), as these lack the structural epitopes required for favorable interactions with *Mp*PA14. For instance, while arabinan is a polymer of arabinose, a sugar that *Mp*PA14 binds, X-ray crystallography showed that *Mp*PA14 is selective in only forming a complex with α-l-arabinopyranose (Fig. 3I). This conformer is not present in the arabinan polymer composed of 1,5-linked α-l-arabino-furanoses, which are then not free to transition to the pyranose form. Similarly, since the *Mp*PA14 binds galactose via the 1,2-diol (β-anomer) (Fig. 3H), the lectin cannot interact with galactans, as they are polymers of β-1,4-d-galactose (Fig. 4B and Table S5).

Some differences were observed in the binding results, with several polysaccharides present in both the first and second glycan microarrays. Examples include mannan from Saccharomyces cerevisiae, which showed weak binding to *Mp*PA14 in the first array (Fig. 4A, glycan number 12) but had no detectable interaction with the lectin in the second array (Fig. 4B; glycan number 8; pullulan bound *Mp*PA14 more strongly than pustulan in the first array (Fig. 4A; glycan numbers 2 and 11, respectively), whereas their relative affinities were reversed in the second array (Fig. 4B; glycan numbers 12 and 20, respectively. These minor discrepancies in the binding results could be due to differences in the polysaccharide sources or the different methods used to immobilize them onto the microarrays (44, 45). Nevertheless, both arrays pointed to the key result that *Mp*PA14 selectively binds glucans with multiple unoccupied 3,4-diols, while the lectin does not recognize glycans such as arabinans, galactans, and xylans. Furthermore, the finding that *Mp*PA14 interacted strongly with fucoidans is consistent with the *Mp*PA14-fucose interaction demonstrated by the binding and structural data. This result may have physiological relevance, as l-fucose-containing polysaccharides are highly prevalent in the exudates of diatoms (46–49), at least one of which is a natural host of M. primoryensis.

Since the PA14 domain is widespread in bacteria, including some that are human commensals and others that are pathogens, we reasoned that *Mp*PA14 and its homologs might interact with mammalian glycans. Therefore, we tested the lectin on a microarray consisting of 609 complex mammalian glycans (Consortium for Functional Glycomics, version 5.2). Four glycans (506, 431, 433, and 474) stood out among the strongest binders (Fig. 4C). They share an architecture as moderately branched mannose-containing oligomers with a bisecting GlcNAc motif. Each of the four glycans has three to five terminal GlcNAc moieties with 3,4-diols available for complexing *Mp*PA14. Glycans 506 and 474 also have one α-l-fucose moiety linked to the surface-immobilized GlcNAc. Interestingly, the strongest binder, glycan 506, differs from glycan 433 only by the addition of the α-l-fucose, suggesting that this monosaccharide might contribute to the higher affinity of glycan 506 by presenting an extra binding site for the lectin. Glycans with fewer binding epitopes of GlcNAc and fucose bound more weakly in general. Out of the 61 glycans that did not bind *Mp*PA14 (relative fluorescence units [RFUs] of 20 or below), 33 have *N*-acetyl-neuraminic acid as their terminal sugar, while another 25 of these nonbinders end with either galactose or GalNAc (Fig. 4C). Indeed, as shown by our structural analyses above, these sugars lack the 3,4-diol motifs of glucopyranoses and l-fucose preferred for *Mp*PA14 recognition.

### l-Fucose blocks PA14-diatom interactions.

Having identified l-fucose as the strongest monosaccharide ligand for *Mp*PA14, we set out to validate its potential as an inhibitor for the lectin-dependent bacteria-diatom interaction that led to the discovery and characterization of this protein (21). Here, we tested if l-fucose can block fluorescently labeled *Mp*PA14 from binding to the diatom Chaetoceros neogracile.

**FIG 5:**
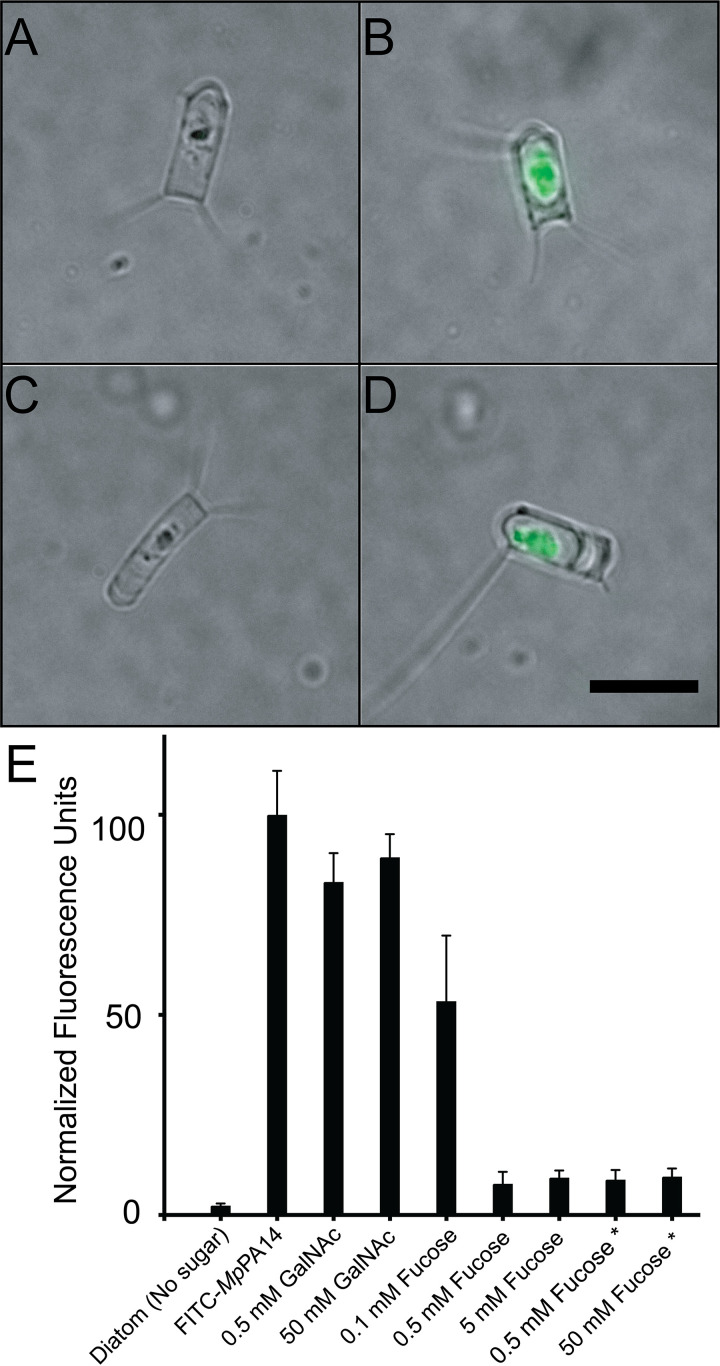
Inhibition of *Mp*PA14 binding to the diatom *C. neogracile*. Representative images showing (A) an untreated *C. neogracile* diatom with basal autofluorescence in the center from its cell; (B) a *C. neogracile* cell treated with 0.2 mg/ml fluorescein isothiocyanate (FITC)-labeled *Mp*PA14; (C) a *C. neogracile* cell treated with 0.5 mM l-fucose and 0.2 mg/ml FITC-labeled *Mp*PA14; and (D) a *C. neogracile* cell treated with 0.2 mg/ml FITC-labeled *Mp*PA14 and 50 mM GalNAc. All four images (A to D) are at the same scale, with the black scale bar in panel D indicating 10 μm. (E) Fluorescence levels shown by the *C. neogracile* cells alone and those treated with various ligands. Each bar represents the quantification of average fluorescence from 30 individual diatoms. Data bars with an asterisk underneath represent experiments where diatoms were incubated with FITC-*Mp*PA14 before l-fucose was added.

C. neogracile is a psychrophilic marine diatom found in Antarctic waters (50). As shown in Fig. 5A and D, *C. neogracile* diatoms are roughly 10 μm in length with a width of 3 to 4 μm. Each diatom cell has 1 to 4 projections protruding from the corners. Given its photosynthetic capability, *C. neogracile* contains chlorophyll that is intrinsically fluorescent. However, the binding of fluorescein isothiocyanate (FITC)-labeled *Mp*PA14 to *C. neogracile* resulted in a 40-fold increase of fluorescence over the basal autofluorescence of the diatom (Fig. 5A and B; see also Fig. S6 in the supplemental material). The addition of 0.5 mM l-fucose was extremely effective at blocking accumulation of lectin on the diatom (Fig. 5C), as the free sugar outcompeted the cell surface glycans for the binding *Mp*PA14 and displaced 95% of the fluorescent signal (Fig. 5E). This competitive effect fell off to ∼40% as the l-fucose concentration was reduced to 0.1 mM (Fig. 5E). The effective concentration of l-fucose needed to block association is significantly higher than the *K_d_* of 147 μM for *Mp*PA14-fucose interaction determined by ITC. We reason that glycans coating the diatom cell membrane present numerous end group binding sites in close proximity that can serve as a “molecular Velcro” for *Mp*PA14 binding (30).

10.1128/mBio.00130-21.6 FIG S6 Representative images of diatoms with various fluorescein isothiocyanate (FITC)-*Mp*PA14 and sugar treatments shown in Fig. 5. Asterisks (*) represent experiments where diatoms were incubated with FITC-*Mp*PA14 before l-fucose was added. Download FIG S6, PDF file, 0.1 MB. Copyright © 2021 Guo et al. 2021 Guo et al. https://creativecommons.org/licenses/by/4.0/ This content is distributed under the terms of the Creative Commons Attribution 4.0 International license.

In contrast to the inhibitory effect of l-fucose, the nonbinder of *Mp*PA14, GalNAc, was unable to prevent the lectin from binding the diatom even at 50 mM (Fig. 5D and E and Fig. S6), validating the results from the binding and structural studies. Importantly, adding 0.5 mM l-fucose to diatoms precoated with FITC-labeled *Mp*PA14 resulted in the dissociation of the lectin from the cells. These results suggest that l-fucose can disrupt preexisting associations between bacteria and diatoms.

### *Mp*PA14 lectin homologs are found in pathogens.

The binding conformations of various monosaccharides identified in the structural analyses can lay the foundation for the structure-guided design of glycan-based probes for detecting microbes or for making inhibitors to disrupt bacterial adhesion. *Mp*PA14 homologs are widespread in the adhesins of Gram-negative bacteria such as the previously reported *Mh*PA14 from the oil-degrading M. hydrocarbonoclasticus, as well as those adhesins that help pathogenic bacteria infect specific niches. For example, a large RTX adhesin from the cholera-causing human pathogen, Vibrio cholerae, contains a *Mp*PA14 homolog (*Vc*PA14) with 44% identity at the protein level. Moreover, the amino acid residues involved in coordinating Ca1 and recognizing glycans are conserved between the *Mp*PA14 and *Vc*PA14 (see Fig. S1G in the supplemental material). Despite small deviations in amino acid sequence between *Mp*PA14 and *Mh*PA14 from *M. hydrocarbonoclasticus*, these lectins have the same monosaccharide ligands, and there is insignificant variation in their complex glycan recognition (12). With its ligand-binding site even more like *Mp*PA14 than that of *Mh*PA14, *Vc*PA14 probably binds to the same simple sugars. It is therefore of interest to test the inhibitory effect of strong PA14 binders identified in this study to set the stage for developing novel strategies for modulating bacterial adhesion.

**FIG 6:**
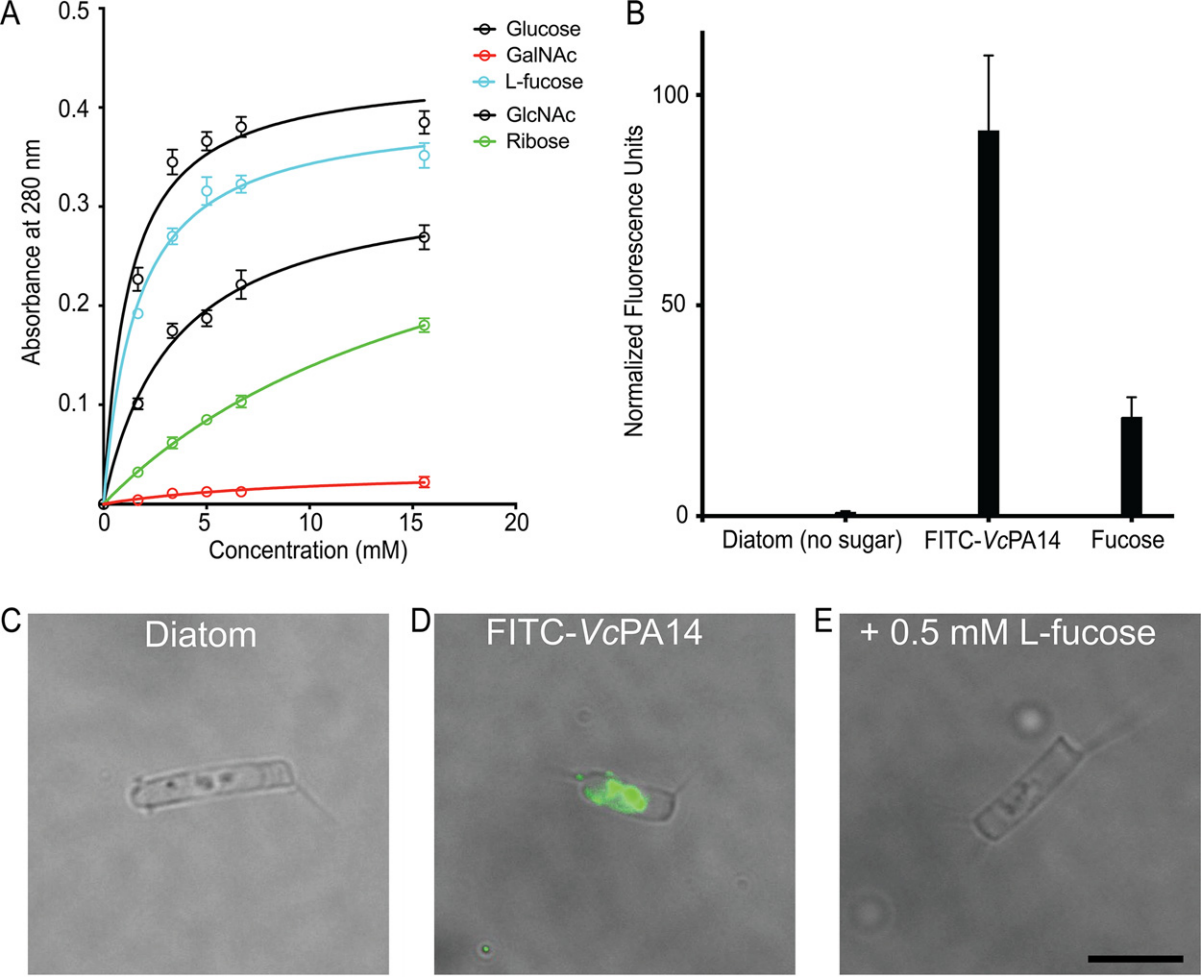
Sugar selectivity of *Vc*PA14 and its interactions with *C. neogracile*. (A) Dextran-based competition binding assay to determine relative affinity of *Mp*PA14 for glucose, GlcNAc, l-fucose, ribose, and GalNAc. (B) Fluorescence levels shown by the *C. neogracile* cells alone and those treated with FITC-*Vc*PA14 in the absence or presence of 0.5 mM l-fucose. Each bar represents the quantification of normalized average fluorescence from 30 individual diatoms. (C to E) Representative images of diatoms alone (C) and those incubated with FITC-*Vc*PA14 in the absence (D) and presence of 0.5 mM l-fucose (E).

As proof of concept for the antiadhesion strategy that targets microbial pathogens, we studied the *Vc*PA14 lectin domain from a large 6,938-amino-acid RTX adhesin (GenBank accession number WP_154597608) of the cholera-causing bacterium, V. cholerae. As discussed, *Vc*PA14 has a similar ligand-binding site as that of *Mp*PA14 (Fig. S1), with key sugar-binding residues conserved (Fig. S1G). Consistent with our structural and functional studies on *Mp*PA14, *Vc*PA14 interacts with GlcNAc, and fucose more strongly than glucose, while the lectin cannot bind GalNAc (Fig. 6A). Moreover, FITC-labeled *Vc*PA14 bound to the cell membrane of *C. neogracile* within the frustule in the same way as *Mp*PA14 did, and this binding was largely blocked by addition of 0.5 mM fucose (Fig. 6B to E).

### Conclusions and outlook.

In this study, we elucidated the molecular basis for ligand recognition by a lectin module widespread in bacterial adhesins. The atomic details revealed by X-ray crystallography not only helped clarify the plasticity of *Mp*PA14 in binding various monosaccharide ligands but also revealed how the lectin recognizes complex polysaccharides in a more specific manner. We further show that a low millimolar amount of l-fucose can be used to disrupt binding of the lectin to diatom cells. The atomic details of the lectin-carbohydrate interactions elucidated here serve as the starting points for the development of adhesin antagonists via ligand-based design. For instance, the fact that GlcNAc gains a 2-fold higher affinity for *Mp*PA14 than glucose simply from the replacement of the C-2 hydroxyl with an acetyl group suggests that appending designed substituents on various positions of avid binders such as the C-2 and C-5 of l-fucose might further enhance their potency (Fig. 2C and F).

Given the high similarity between *Mp*PA14 and lectin folds in the adhesins of pathogenic bacteria, this work gives insight into how harmful bacterium-host interactions might be controlled through modulation of the lectin-glycan interactions. This antiadhesion approach holds promise as an alternative or additive approach to treat infections without the excessive use of antibiotics and may thus help mitigate problems with multidrug-resistant bacteria (17, 51, 52).

## MATERIALS AND METHODS

### Dextran-based comparative competition assay.

The dextran resin competition assay was performed as previously described for *Mh*PA14 (12). Briefly, Superdex 200 (S200) resin was washed twice with 50 mM Tris-HCl (pH 9), 150 mM NaCl, and 5 mM CaCl_2_. One ml of 1 mg/ml *Mp*PA14 with green fluorescent protein (GFP) fused to its N terminus (GFP-*Mp*PA14) or *Vc*PA14 labeled with FITC was suspended with 300 μl of equilibrated Superdex 200 (S200) resin. Following an incubation period with gentle mixing, the S200 resin bound with GFP-*Mp*PA14 was pelleted by centrifugation. The pellet was washed twice with 50 mM Tris-HCl (pH 9), 150 mM NaCl, and 5 mM CaCl_2_, and the *A*_280_ of the supernatant from the second wash was used as the baseline reading. Next, after resuspension in the same buffer, aliquots of 1.67 μmol saccharide were sequentially added to the solution six or seven times, with the *A*_280_ of the supernatant being measured after each addition to quantify the release of lectin. The final addition of saccharide was 5 μM. Data from the dextran affinity assay were plotted using GraphPad Prism after subtracting the background. Next, the data were fitted to a nonlinear regression of one-site-specific binding, which follows the model *y/B*_max_ = *x*/(*K_d_* + *x*), with *B*_max_ as the maximum specific binding and *K_d_* as the equilibrium binding constant.

### Isothermal titration calorimetry.

Isothermal titration calorimetry (ITC) measurements were performed at 30°C with a MicroCal VP-ITC instrument (Malvern). *Mp*PA14 (400 μM) was mixed with serial 5-μl aliquots of 8 mM sugar solution (l-fucose, GlcNAc, or glucose). Sugars were automatically added by a rotating syringe (400 rpm) at 5-min intervals into the *Mp*PA14 solution for a total of 50 injections. The data were analyzed with Origin software version 5.0 (MicroCal).

### Cocrystallization, X-ray diffraction, and structure solutions of *Mp*PA14 with various sugars.

Details for the cloning, expression, purification, and crystallization of *Mp*PA14 were previously reported (12, 21). Cocrystallization of *Mp*PA14 with various sugars was performed using the “microbatch-under-oil” method by mixing equal volumes of ∼20 mg/ml protein with a precipitant solution composed of 0.2 M calcium chloride, 0.1 M HEPES (pH 7), 20% (vol/vol) polyethylene glycol 3350, and 0.5 to 1 M different sugars. In addition to the previously reported *Mp*PA14-glucose structure, the 14 different new sugars that were complexed with *Mp*PA14 were l-fucose, GlcNAc, galactose, allose, mannose, 3-*O*-methyl-glucose, 2-deoxy-glucose, α-methyl-glucose, myo-inositol, sucrose, trehalose, l-arabinose, ribose, and 2-deoxy-ribose.

X-ray crystallographic data were collected at either the 08ID-1 beamline of the Canadian Light Source synchrotron facility or at the 23-ID-B beamline of the Advanced Photon Source via remote access. Data were indexed and integrated with X-ray Detector Software (XDS) (53) and CCP4-Aimless (54) or the DIALS/xia2 in the CCP4i2 software suite (55). The structure solutions for all complexes were obtained by molecular replacement using the *Mp*PA14 glucose-bound structure as the search model (21). The structures were refined using CCP4-Refmac5 (56) or Phenix (57).

### Glycan arrays.

Three different glycan arrays were probed with *Mp*PA14. Two of the arrays focused on fungal, bacterial, algal, and plant polysaccharides, and the other on mammalian glycans. The first glycan array was done at the Carbohydrate Microarray Facility (Glycosciences Laboratory, Imperial College). GFP-*Mp*PA14 (50 μg/ml) was exposed to the “Fungal, bacterial and plant polysaccharide array set 2,” which contained duplicates of 20 saccharide probes from a variety of organisms. An Alexa Fluor 647-tagged anti-GFP antibody was used for detecting the lectin, and the duplicates were averaged to produce the final relative fluorescence unit (RFU) values. In a negative-control experiment, where anti-GFP antibody was directly reacted to the saccharide probes, four glycans, namely lipomannan and lipoarabinomannan from Mycobacterium tuberculosis, lipoarabinomannan from Mycobacterium smegmatis, and native *O*-glycoprotein from M. tuberculosis, showed significant binding to the anti-GFP antibody, as they produced RFUs of greater than 1,000. This indicated that these four glycan samples yielded false-positive results. Therefore, these four polysaccharides were discarded from the analyses shown in Results of our paper.

The second glycan array was performed at the Max Planck Institute for Marine Microbiology (Bremen, Germany). The array contained duplicates of 32 polysaccharides, including those from macroalgae, bacteria, fungi, and land plants (see details in Table S5 in the supplemental material). N-terminally His-tagged *Mp*PA14 was incubated with the array, and binding of the lectin to the polysaccharides was detected by an anti-His tag secondary antibody conjugated to alkaline phosphatase (Sigma-Aldrich). Microarray probing and quantification were performed as previously described (45). Maximal mean (average of the duplicates) signal intensity was set to 100, and the rest of values were normalized accordingly. A cutoff of 5 was applied (58).

The third glycan array screening was done by the Consortium for Functional Glycomics (Harvard Medical School) using version 5.2 of a printed mammalian glycan array, which contained 609 glycans (59). Tetramethyl rhodamine isocyanate (TRITC)-labeled *Mp*PA14 was incubated with the surface-immobilized glycans, and the array was scanned at an excitation wavelength of 532 nm. The resulting RFUs were used as a measure of the bound protein. Each glycan was present in six replicates on the array, and the highest and lowest value from each set was omitted to avoid outlying values. The RFU values from the remaining four replicates were averaged.

### Diatom binding experiments.

The Antarctic diatom, Chaetoceros neogracile, was cultured as previously described (21, 50). FITC-labeled *Mp*PA14 or *Vc*PA14 (FITC-*Mp*PA14 or FITC-*Vc*PA14, 0.2 mg/ml) in the presence or absence of sugars was incubated with diatoms in buffer (50 mM Tris-HCl [pH 9], 300 mM NaCl, and 5 mM CaCl_2_) with gentle mixing for 2 h. Next, diatoms were pelleted by centrifugation for 3 min at 7,000 rpm, and the resulting supernatant was discarded. This procedure was repeated three times to wash away unbound FITC-*Mp*PA14 before the diatom pellet was finally resuspended in 20 μl buffer, which was then used to make slides for fluorescence microscopy. In a separate experiment to test if fucose could compete off the *Mp*PA14 that was already bound to diatoms, FITC-*Mp*PA14 was incubated with diatom for 1.5 h before fucose was added. The rest of the experiment followed the same procedure as described above.

Images were obtained using an Olympus IX83 inverted fluorescence microscope equipped with an Andor Zyla 4.2 Plus camera. Quantification of the fluorescence intensity was done using Fiji ImageJ. The corrected total cell fluorescence (CTCF) was calculated using the following formula: CTCF = integrated density − (area of selected cell × mean fluorescence of the background) (60). Quantification of 30 individual diatom cells was done for each treatment. Graphs were made using GraphPad Prism.

### Data availability.

X-ray crystal structure coordinates solved in this study have been deposited in the Protein Data Bank under accession codes 6X7J (*Mp*PA14-fucose), 6X7X (*Mp*PA14-mannose), 6XAQ (*Mp*PA14-α-methyl-glucose), 6X7Z (*Mp*PA14-inositol), 6X7Y (*Mp*PA14-GlcNAc), 6X7T (*Mp*PA14-allose), 6X9M (*Mp*PA14-3-*O*-methyl-glucose), 6X95 (*Mp*PA14-2-deoxy-glucose), 6XAC (*Mp*PA14-galactose), 6X8D (*Mp*PA14-arabinose), 6X8Y (*Mp*PA14-ribose), 6X9P (*Mp*PA14-2-deoxy-ribose), 6X8A (*Mp*PA14-sucrose), and 6XA5 (*Mp*PA14-trehalose). The data that support the findings of this study are available from the corresponding author, P. L. Davies, upon reasonable request.
