# Supplementary material for: Structural Basis of Ligand Selectivity by a Bacterial Adhesin Lectin Involved in Multispecies Biofilm Formation
Source: mBio. 2021 Apr 6;12(2):e00130-21. doi: 10.1128/mBio.00130-21 (PMC8092209; doi:10.1128/mBio.00130-21)
Supplement: TABLE S1 [file mBio.00130-21-st001.docx]

**Table S1:** X-ray crystallographic statistics for *Mp*PA14 in complex with L-fucose, mannose, α-methyl-Glucose, Inositol, GlcNAc, allose and 3-o-methyl glucose.

| **Data collection** | **L-fucose** | **Mannose** | **α-methyl-Glc** | **Inositol** | **GlcNAc** | **Allose** | **3-O-methyl glucose** |
| --- | --- | --- | --- | --- | --- | --- | --- |
| **PDB code** | 6X7J | 6X7X | 6XAQ | 6X7Z | 6X7Y | 6X7T | 6X9M |
| Space group | P 21 21 21 | P 21 21 21 | P 21 21 21 | P 21 21 21 | P 21 21 21 | P 21 21 21 | P 21 21 21 |
| **Cell dimensions** |  |  |  |  |  |  |  |
| (a, b, c) (Å) | 45.08 50.81 79.49 | 45.30, 50.36, 79.13 | 45.12, 50.50, 79.39 | 45.15, 50.57, 79.62 | 45.24 50.65 79.42 | 45.47, 50.27, 79.01 | 45.2, 50.33, 79.14 |
| (α, β, γ) (°) | 90.0, 90.0, 90.0 | 90.0, 90.0, 90.0 | 90.0, 90.0, 90.0 | 90.0, 90.0, 90.0 | 90.0, 90.0, 90.0 | 90.0, 90.0, 90.0 | 90.0, 90.0, 90.0 |
| Resolution (Å) | 42.81 - 0.97 | 79.1 - 1.26 | 50.5 - 0.94 | 79.6 - 0.92 | 39.7-0.96 | 42.41 - 0.96 | 42.47 - 0.97 |
| No. of observations | 1175389 | 377043 | 2088235 | 1201596 | 1182800 | 1185822 | 1129177 |
| No. of unique | 100735 | 49044 | 100057 | 111883 | 106951 | 106122 | 101513 |
| No. molecules/asymmetric unit | 1 | 1 | 1 | 1 | 1 | 1 | 1 |
| I/σI | 17.9 (0.8) | 4.79 (1.22) | 17.83 (1.59) | 23.44 (1.63) | 17.1 (1) | 23.1 (2) | 19.9 (1) |
| R_merge_ | 0.076 (1.9) | 0.292 (0.620) | 0.091 (0.901) | 0.057 (0.560) | 0.06(1.03) | 0.058 (0.64) | 0.058 (1.19) |
| CC(1/2) | 0.99 (0.36) | 0.95 (0.46) | 0.99 (-0.12) | 0.99 (0.25) | 0.99 (0.49) | 0.99 (0.76) | 0.99 (0.57) |
| Completeness (%) | 91.8 (63.7) | 98.8 (83.6) | 84.5 (13.4) | 88.6 (17.2) | 96.1 (70) | 95.6 (66.2) | 94.6 (59.8) |
| Multiplicity | 11.7 (5.2) | 7.7 (3.7) | 20.9 (1.7) | 10.7 (1.8) | 11.1 | 11.2 | 11.1 |
| **Refinement** |  |  |  |  |  |  |  |
| Resolution (Å) | 39.7 - 0.97 | 39.6 - 1.3 | 42.6 - 1.2 | 42.7 - 1 | 39.7 - 1 | 42.4 - 1 | 42.5 - 1 |
| R_work_/ R_free_ (%) | 12.1/13.7 | 15.8/18.6 | 16.3/16.7 | 12.5/13.1 | 12.7/14.1 | 12.7/13.0 | 14.2/15.9 |
| **No. of atoms** |  |  |  |  |  |  |  |
| protein/Ion/ligand/ water | 2824/7/54/352 | 1576/9/50/265 | 2743/8/27/262 | 2702/6/82/274 | 2666/4/78/252 | 2772/6/48/282 | 2679/5/73/301 |
| **B-factors (Å^2^)** |  |  |  |  |  |  |  |
| protein/Ion/ligand/water | 8.3/8.3/10.1/21.7 | 7.5/13.4/15.2/23.8 | 11.0/12.7/14.8/22.5 | 8.5/9.6/12.6/19.5 | 10.3/9.1/14.2/21.5 | 9.6/7.8/21.0/24.7 | 10.9/8.0/20.0/23/5 |
| **r.m.s deviations** |  |  |  |  |  |  |  |
| Bond lengths (Å) | 0.015 | 0.014 | 0.013 | 0.014 | 0.016 | 0.011 | 0.009 |
| Bond angles (°) | 1.615 | 1.666 | 1.55 | 1.987 | 1.682 | 1.45 | 1.321 |
| **Ramachandron statistics** |  |  |  |  |  |  |  |
| Favored | 96.11 | 94.05 | 95.56 | 96.09 | 96.11 | 96.11 | 96.11 |
| Outliers | 0.56 | 0.54 | 0.56 | 0.56 | 0.56 | 0.56 | 0.56 |
