# Supplementary figures and images for: Structural Basis of Ligand Selectivity by a Bacterial Adhesin Lectin Involved in Multispecies Biofilm Formation
Source: mBio. 2021 Apr 6;12(2):e00130-21. doi: 10.1128/mBio.00130-21 (PMC8092209; doi:10.1128/mBio.00130-21)

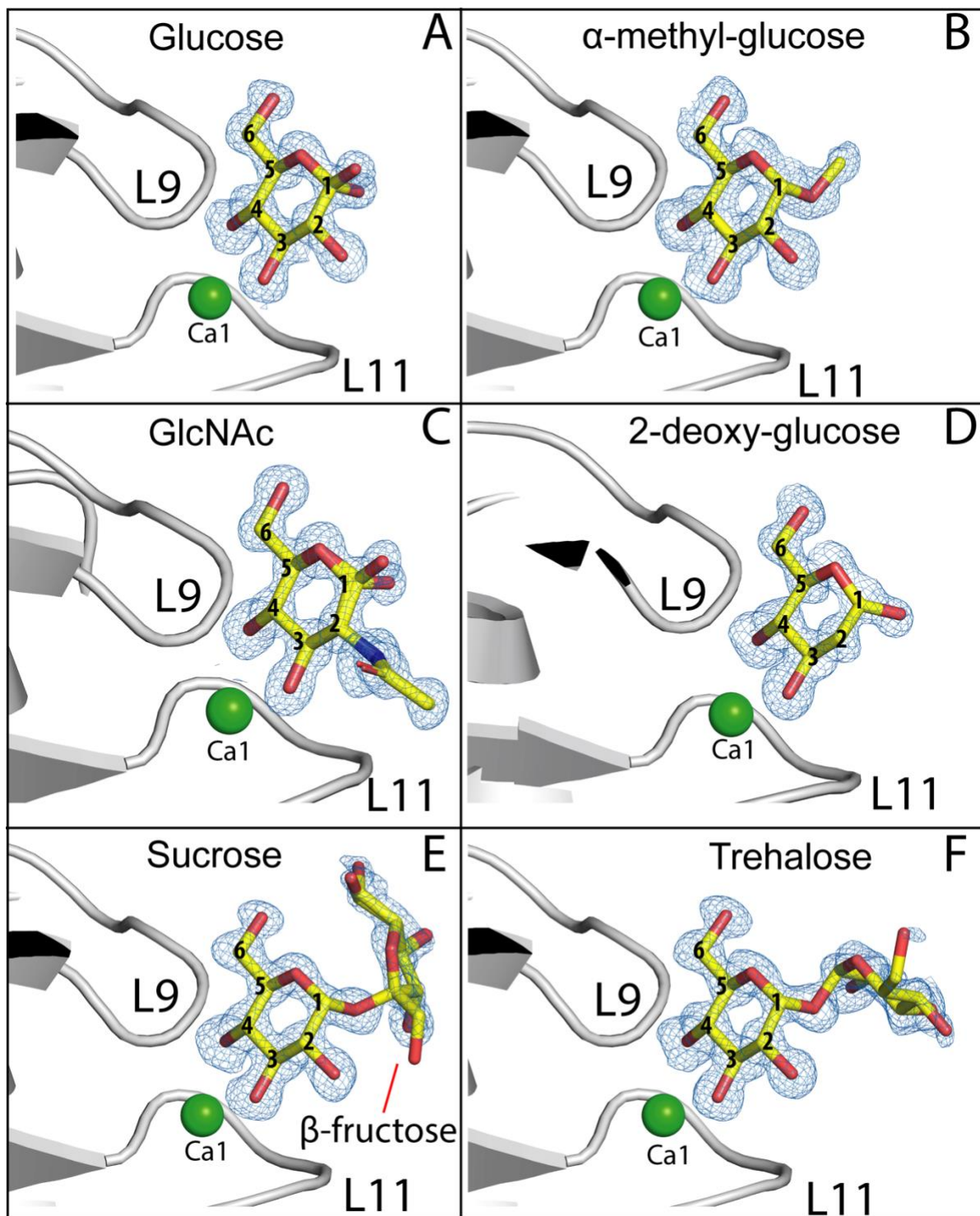

Supplement: FIG S2 [file mBio.00130-21-sf002.pdf]

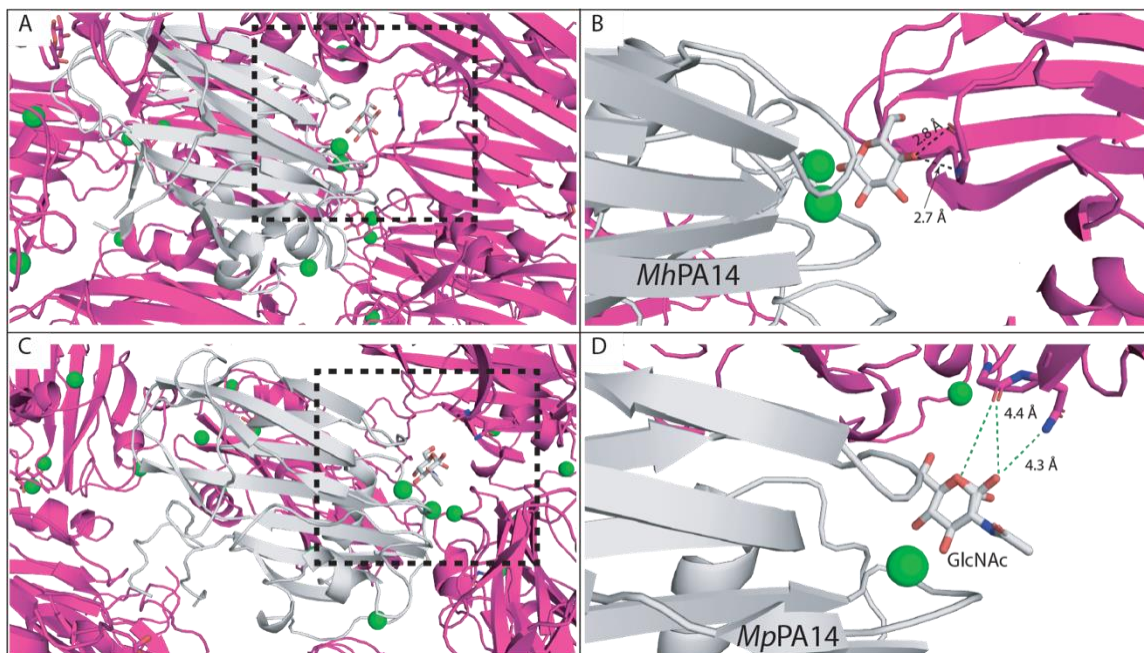

Supplement: FIG S4 [file mBio.00130-21-sf004.pdf]

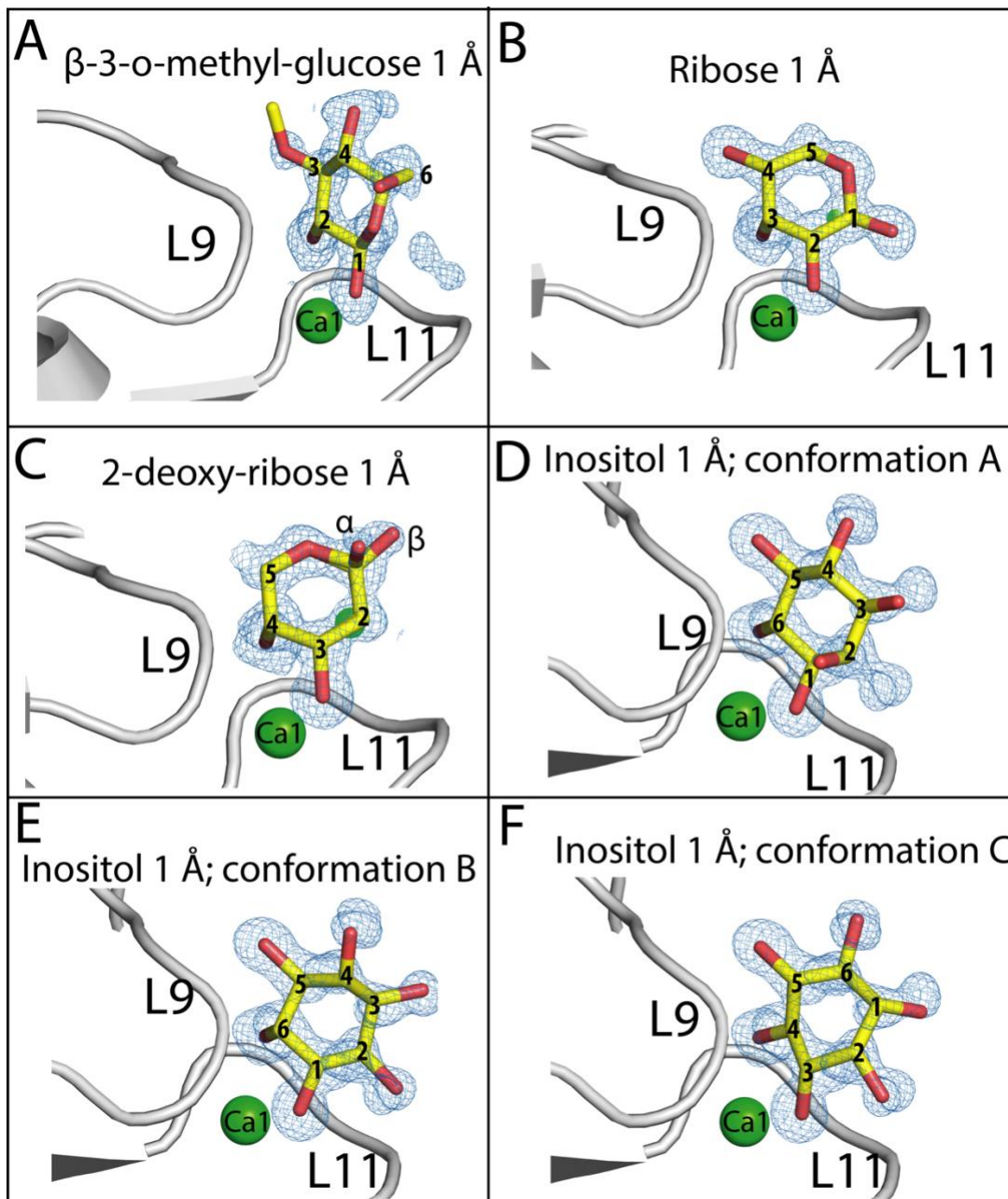

Supplement: FIG S5 [file mBio.00130-21-sf005.pdf]

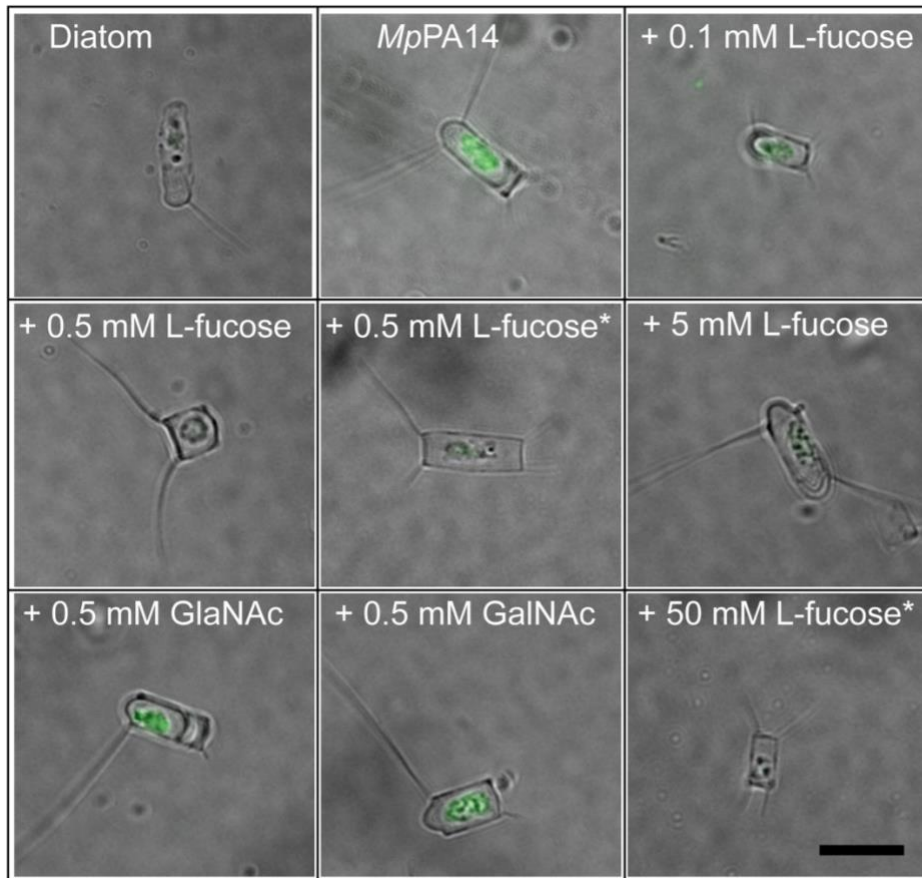

Supplement: FIG S6 [file mBio.00130-21-sf006.pdf]
