# Supplementary material for: Structural Basis of Ligand Selectivity by a Bacterial Adhesin Lectin Involved in Multispecies Biofilm Formation
Source: mBio. 2021 Apr 6;12(2):e00130-21. doi: 10.1128/mBio.00130-21 (PMC8092209; doi:10.1128/mBio.00130-21)
Supplement: FIG S3 [file mBio.00130-21-sf003.pdf]

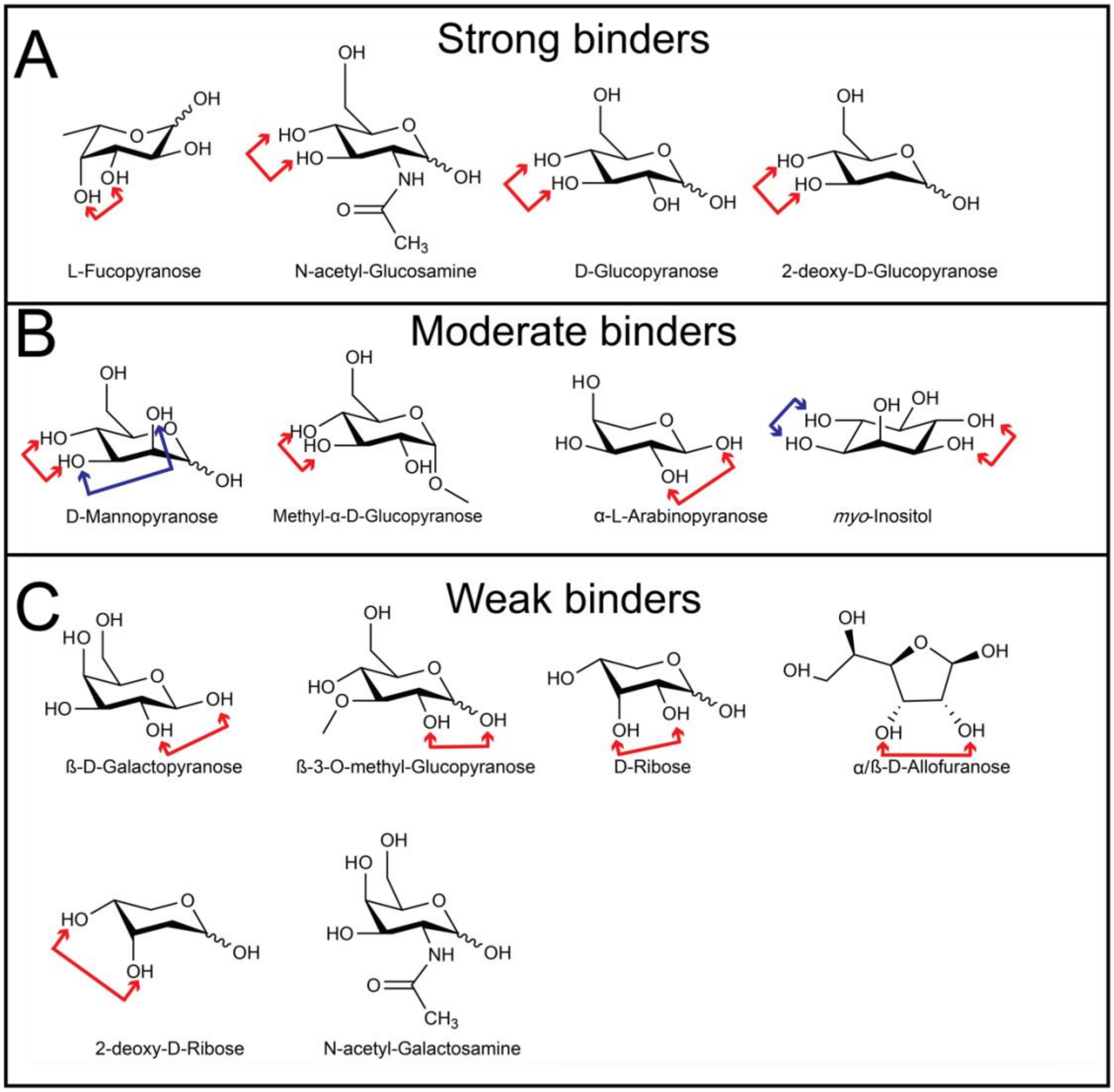
A
Strong binders
L-Fucopyranose
N-acetyl-Glucosamine
D-Glucopyranose
2-deoxy-D-Glucopyranose
B
Moderate binders
D-Mannopyranose
Methyl-α-D-Glucopyranose
α-L-Arabinopyranose
myo-Inositol
C
Weak binders
ß-D-Galactopyranose
ß-3-O-methyl-Glucopyranose
D-Ribose
α/ß-D-Allofuranose
2-deoxy-D-Ribose
N-acetyl-Galactosamine
