# Supplementary material for: Structural Basis of Ligand Selectivity by a Bacterial Adhesin Lectin Involved in Multispecies Biofilm Formation
Source: mBio. 2021 Apr 6;12(2):e00130-21. doi: 10.1128/mBio.00130-21 (PMC8092209; doi:10.1128/mBio.00130-21)
Supplement: TABLE S2 [file mBio.00130-21-st002.docx]

**Table S2:** X-ray crystallographic statistics for *Mp*PA14 in complex with 2-deoxy-glucose, galactose, L-arabinose, ribose, 2-deoxy-ribose, sucrose and trehalose.

| **Data collection** | **2-deoxy-glucose** | **Galactose** | **L-arabinose** | **Ribose** | **2-deoxy ribose** | **Sucrose** | **Trehalose** |
| --- | --- | --- | --- | --- | --- | --- | --- |
| **PDB code** | 6X95 | 6XAC | 6X8D | 6X8Y | 6X9P | 6X8A | 6XA5 |
| Space group | P 21 21 21 | P 21 21 21 | P 21 21 21 | P 21 21 21 | P 21 21 21 | P 21 21 21 | P 21 21 21 |
| **Cell dimensions** |  |  |  |  |  |  |  |
| (a, b, c) (Å) | 45.19, 50.03, 77.79 | 45.24 50.04 78.71 | 45.12, 50.71, 79.62 | 45.14, 50.72, 79.61 | 45.22, 50.53, 79.47 | 45.23 50.61 79.60 | 45.27 50.64 79.43 |
| (α, β, γ) (°) | 90.0, 90.0, 90.0 | 90.0, 90.0, 90.0 | 90.0, 90.0, 90.0 | 90.0, 90.0, 90.0 | 90.0, 90.0, 90.0 | 90.0, 90.0, 90.0 | 90.0, 90.0, 90.0 |
| Resolution (Å) | 42.08 - 0.96 | 45.22 - 1.22 | 42.77 - 0.96 | 42.78 - 0.96 | 39.74 - 0.96 | 42.71 - 1.06 | 42.7 - 1.03 |
| No. of observations | 1130090 | 370846 | 1171516 | 1152123 | 1182113 | 499930 | 564807 |
| No. of unique | 103370 | 51321 | 106425 | 103936 | 106580 | 61637 | 75689 |
| No. molecules/asymmetric unit | 1 | 1 | 1 | 1 | 1 | 1 | 1 |
| I/σI | 17.1 (0.2) | 28.9 (8.8) | 19.1 (0.9) | 10.1 (0.3) | 17.8 (0.4) | 15.3 (1.2) | 28.0 (2.6) |
| R_merge_ | 0.055 (5.8) | 0.044 (0.092) | 0.064 (1.6) | 0.14 (6.3) | 0.06 (2.8) | 0.11 (1.2) | 0.047 (0.34) |
| CC(1/2) | 0.99 (0.12) | 0.99 (0.98) | 0.99 (0.62) | 0.99 (0.33) | 0.99 (0.12) | 0.99 (0.28) | 0.99 (0.88) |
| Completeness (%) | 96.3 (67.7) | 95.1 (65.9) | 95.2 (62.7) | 92.8 (47.3) | 95.8 (65.0) | 74.6 (11) | 84.2 (15.6) |
| Multiplicity | 10.9 | 7.2 | 11 | 1.1 | 11.1 | 8.1 | 7.5 |
| **Refinement** |  |  |  |  |  |  |  |
| Resolution (Å) | 42.1 - 1.05 | 42.2 - 1.22 | 42.8 - 1 | 42.8 - 1 | 39.7 - 1 | 42.7 - 1.06 | 42.7-1.03 |
| R_work_/ R_free_ (%) | 16.0/17.3 | 13.4/16.0 | 14.6/16.6 | 15.5/16.7 | 12.9/14.2 | 14.2/16.3 | 11.8/13.1 |
| **No. of atoms** |  |  |  |  |  |  |  |
| protein/Ion/ligand/ water | 2656/5/61/287 | 1439/7/12/255 | 2755/6/50/285 | 1391/4/22/283 | 2719/5/78/306 | 1432/7/23/265 | 2748/7/45/290 |
| **B-factors (Å^2^)** |  |  |  |  |  |  |  |
| protein/Ion/ligand/water | 13.0/10.0/30.6/25.1 | 13.7/15.7/14.4/27.0 | 12.8/8.6/24.4/23.2 | 8.5/9.1/17.0/23.8 | 11.0/10.3/22.8/25.2 | 9.4/12.0/22.5/24.2 | 9.4/8.7/20.9/20.9 |
| **r.m.s deviations** |  |  |  |  |  |  |  |
| Bond lengths (Å) | 0.008 | 0.016 | 0.017 | 0.013 | 0.008 | 0.011 | 0.008 |
| Bond angles (°) | 1.173 | 1.689 | 1.672 | 1.535 | 1.222 | 1.442 | 1.224 |
| **Ramachandron statistics** |  |  |  |  |  |  |  |
| Favored | 96.11 | 95.68 | 96.07 | 96.11 | 96.11 | 95.11 | 95.68 |
| Outliers | 0.56 | 1.08 | 0.56 | 0.56 | 0.56 | 1.09 | 1.08 |
