# Supplementary material for: Structural Basis of Ligand Selectivity by a Bacterial Adhesin Lectin Involved in Multispecies Biofilm Formation
Source: mBio. 2021 Apr 6;12(2):e00130-21. doi: 10.1128/mBio.00130-21 (PMC8092209; doi:10.1128/mBio.00130-21)
Supplement: TABLE S3 [file mBio.00130-21-st003.docx]

**Table S3**. Polysaccharides used in the first glycan array. First column on the left presents the relative lectin binding activity detected.

| **Binding of**  **MpPA14 (RFUs)** | **Glycan**  **number** | **Polysaccharide** | **Source** | **Organism** |
| --- | --- | --- | --- | --- |
| 41390 | 1 | Dextran (α-(1→6)-D-glucan backbone) | Leuconostoc mesenteroides | Bacterium |
| 58106 | 2 | Pullulan (α-(1→4)(1→6)-D-glucan) | Pullularia pullulans | Fungus |
| 167 | 3 | Curdlan (β-(1→3)-D-glucan) | Agrobacterium sp. | Bacterium |
| 2319 | 4 | NSG (β-(1→3)-D-glucan backbone, β-(1→3)-D-glucan branches) | Saccharomyces cerevisiae | Fungus |
| 30143 | 5 | PGPGG (β-(1→3)-D-glucan backbone, β-(1→3)-D-glucan branches) | Saccharomyces cerevisiae | Fungus |
| 41060 | 6 | Lentinan (β-(1→3)-D-glucan backbone, β-(1→3)-D-glucan branches) | Lentinula edodes | Fungus |
| 39935 | 7 | Grifolan (β-(1→3)-D-glucan backbone, highly branched oligomers) | Grifola frondosa | Fungus |
| 23 | 8 | β-glucan Mixed β-(1→3)/β-(1→4)-D-glucan | Barley | Plant |
| 0 | 9 | β-glucan Mixed β-(1→3)/β-(1→4)-D-glucan | Oat | Plant |
| 349 | 10 | Lichenan Mixed β-(1→3)/β-(1→4)-D-glucan | Lichen | Fungus |
| 1565 | 11 | Pustulan (β-(1→6)-D-glucan) | Umbilicaria papullosa | Bacterium |
| 3808 | 12 | Mannan (α-(1→6)-D-mannan backbone, α-(1→2)-,α-(1→3)-mannan branches) | Saccharomyces cerevisiae | Fungus |
| 17438 | 13 | N -Mannoprotein (α-1→6-mannan backbone with oligomeric α-(1→2)-, α-(1→3)-, and β-(1→2)-mannan branches | Candida albicans | Fungus |
| 0 | 14 | Mannoprotein (mannose rich) | Aspergillus fumigatus | Fungus |
| 75 | 15 | Glucurono-XyloMannan (α-(1→3)-Mannan with Xyl, GlcA and Fuc branches) | Tremella fuciformis | Fungus |
| 178 | 16 | GN6-AO (chitin, β-1→4-GlcNAc) | - | - |
