# Supplementary material for: Structural Basis of Ligand Selectivity by a Bacterial Adhesin Lectin Involved in Multispecies Biofilm Formation
Source: mBio. 2021 Apr 6;12(2):e00130-21. doi: 10.1128/mBio.00130-21 (PMC8092209; doi:10.1128/mBio.00130-21)
Supplement: TABLE S4 [file mBio.00130-21-st004.docx]

Table S4. Polysaccharides contained in the second glycan array. First column on the left presents the lectin binding activity detected (normalized GFP-*Mp*PA14 signal intensity). Polysaccharide structures are listed in the same order as in x-axis from Fig. 4B (order of appearance shown in second column).

| **Binding of**  **MpPA14 (RFUs)** | **Glycan**  **number** | **Polysaccharide** | **Source** | **Organism** |
| --- | --- | --- | --- | --- |
| 0 | 1 | Polygalacturonic acid (α-(1→4)-D-galacturonic acid) | Citrus pectin | Land plants |
| 0 | 2 | α-(1→5)-L-arabinan | Sugar beet | Land plants |
| 0 | 3 | β-(1→4)-D-galactan | Potato | Land plants |
| 0 | 4 | Rhamnogalacturonan I | Soybean | Land plants |
| 15 | 5 | Mannan (β-(1→4)-D-mannan) | Ivory nut | Land plants |
| 0 | 6 | Galactomannan (β-(1→4)-D-mannan backbone) | Carob | Land plants |
| 0 | 7 | Glucomannan (β-(1→4)-D-mannose and D-glucose backbone) | Konjac | Land plants |
| 0 | 8 | Mannan (α-(1→6)-D-mannan backbone) | Saccharomyces cerevisiae | Yeast (Fungi) |
| 0 | 9 | Glucurono-Xylomannan (α-(1→3)-D-mannan backbone) | Tremella fuciformis | Fungi |
| 0 | 10 | Arabinoxylan | Wheat flour | Land plants |
| 0 | 11 | β-(1→4)-D-xylan | Beechwood | Land plants |
| 37 | 12 | Pullulan (α-(1→4)(1→6)-D-glucan) | Pullularia pullulans | Fungi |
| 28 | 13 | Carboxymethyl cellulose (β-(1→4)-D-glucan) | not available | Land plants |
| 39 | 14 | Laminarin (β-(1→3)-D-glucan backbone with 1→6 branches) | Eisenia bicyclis | Macroalgae |
| 41 | 15 | Laminarin (β-(1→3)-D-glucan backbone with 1→6 branches) | Laminaria digitata | Macroalgae |
| 75 | 16 | Pachyman (β-(1→3)-D-glucan backbone with 1→6 branches) | Poria cocos | Fungi |
| 65 | 17 | Scleroglucan (β-(1→3)-D-glucan backbone with 1→6 branches) | Sclerotium rolfsii | Fungi |
| 0 | 18 | β-glucan (β-(1→3)(1→4)-D-glucan) | Barley | Land plants |
| 44 | 19 | Lichenan (β-(1→3)(1→4)-D-glucan) | Icelandic moss | Lichen (Fungi) |
| 86 | 20 | Pustulan (β-(1→6)-D-glucan) | Lasallia pustulata | Lichen (Fungi) |
| 0 | 21 | Xyloglucan | Tamarind seed | Land plants |
| 34 | 22 | Ulvan | Ulva armoricana | Macroalgae |
| 29 | 23 | Fucoidan | Fucus vesiculosus | Macroalgae |
| 84 | 24 | Fucoidan | Sargassum | Macroalgae |
| 69 | 25 | Fucoidan | Laminaria | Macroalgae |
| 40 | 26 | Galactofucan | Undaria pinnatifida | Macroalgae |
| 100 | 27 | Fucoidan | Cladosiphon | Macroalgae |
| 77 | 28 | Fucoidan | Ascophyllum nodosum | Macroalgae |
| 53 | 29 | Fucoidan | Fucus serratus | Macroalgae |
| 0 | 30 | Partially acetylated alginate | Azotobacter vinelandii | Bacteria |
| 22 | 31 | Alginate | Brown algae | Macroalgae |
| 0 | 32 | Porphyran | Porphyra umbilicalis | Macroalgae |
