# Supplementary material for: Structural Basis of Ligand Selectivity by a Bacterial Adhesin Lectin Involved in Multispecies Biofilm Formation
Source: mBio. 2021 Apr 6;12(2):e00130-21. doi: 10.1128/mBio.00130-21 (PMC8092209; doi:10.1128/mBio.00130-21)
Supplement: FIG S1 [file mBio.00130-21-sf001.pdf]

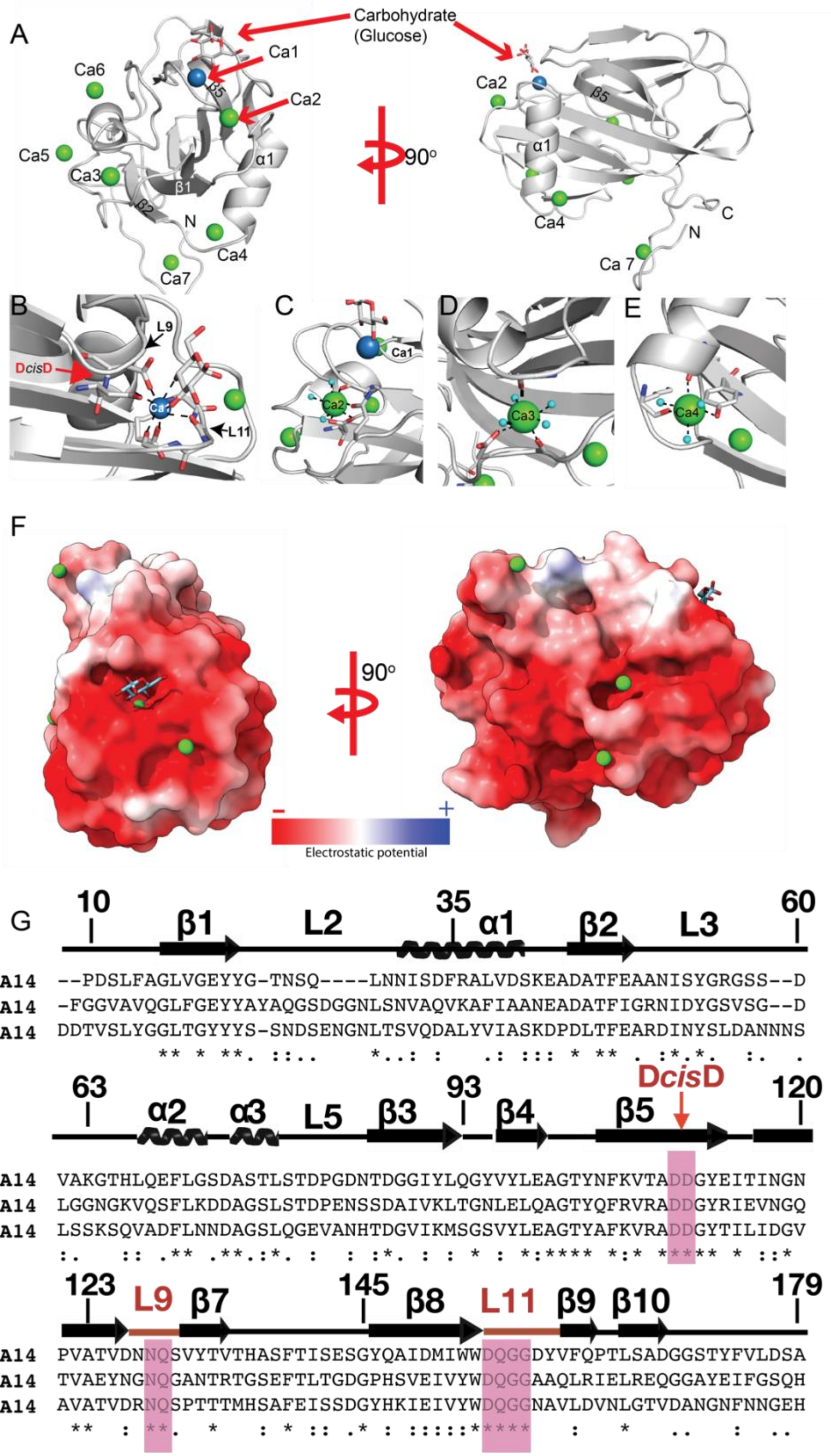
A
Carbohydrate (Glucose)
Ca1
Ca2
Ca3
Ca4
Ca5
Ca6
Ca7
Ca 7
β1
β2
β5
α1
N
C
90°
B
C
D
E
DcisD
L9
L11
F
Electrostatic potential
G
10
35
60
63
93
120
123
145
179
β1 L2 α1 β2 L3
α2 α3 L5 β3 β4 β5
L9 β7 β8 L11 β9 β10
MpPA14 --PDSLFAGLVGEYYG-TNSQ----LNNISDFRALVDSKEADATFEAANISYGRGSS--D
MhPA14 -FGGVAVQGLFGEYYAYAQGSDGGNLSNVAQVKAFIAANEADATFIGRNIDYGSVSG--D
VcPA14 DDTVSLYGGLTGYYYS-SNDSENGNLTSVQDALYVIASKDPDLTFEARDINYSLDANNNS
MpPA14 VAKGTHLQEFLGSDASTLSTDPGDNTDGGIYLQGYVYLEAGTYNFKVTADDGYEITINGN
MhPA14 LGGNGKVQSFLKDDAGSLSTDPENSSDAIVKLTGNLELQAGTYQFRVRADDGYRIEVNGQ
VcPA14 LSSKSQVADFLNNDAGSLQGEVANHTDGVIKMSGSVYLEAGTYAFKVRADDGYTILIDGV
MpPA14 PVATVDNNQSVYTVTHASFTISESGYQAIDMIWWDQGGDYVFQPTLSADGGSTYFVLDSA
MhPA14 TVAEYNGNQGANTRTGSEFTLTGDGPHSVEIVYWDQGGAAQLRIELREQGGAYEIFGSQH
VcPA14 AVATVDRNQSPTTTMHSAFEISSDGYHKIEIVYWDQGGNAVLDVNLGTVDANGNFNNGEH
